# Hazardous Chemicals Road Transportation Accidents and the Corresponding Evacuation Events from 2012 to 2020 in China: A Review

**DOI:** 10.3390/ijerph192215182

**Published:** 2022-11-17

**Authors:** Weihua Zhang, Wuyi Cheng, Wenmei Gai

**Affiliations:** School of Engineering and Technology, China University of Geosciences (Beijing), Beijing 100083, China

**Keywords:** accident statistics, hazardous chemicals transport, evacuation scenario, evacuation event classification, evacuation capacity

## Abstract

Based on accident data from the China Chemical Accident Information Network, detailed information was obtained from 2657 hazardous chemicals road transportation accidents (HCRTAs) and 148 evacuations caused by these accidents that occurred in China from 2012 to 2020. The characteristics and the development trend of the present HCRTAs in China and the rate of emergency are obtained via statistical analysis. Based on the probability of evacuation scenarios via historical statistics, the social cost of labor loss value of participating emergency responders, and evacuees’ placement and transfer cost as the consequences of evacuation events, an evacuation event grading model based on social risk assessment is constructed. Evaluating and classifying the risk of evacuation events caused by HCRTAs (148), the results demonstrated that the social risk caused by emergency scenarios F_61 (leakage due to overturning of hazardous chemical vehicles, which led to evacuation) and F_91 (leakage due to rear-end of hazardous chemical vehicles, which led to evacuation) was higher than other emergency scenarios. To reduce the dangers caused by HCRTAs, the framework for improving the emergency response capacity of communities is discussed and analyzed based on five aspects, which comprise land use planning, city construction, education promotion, information construction, and the layout of emergency resources.

## 1. Introduction

China’s petroleum and chemical industry has developed rapidly in recent years, and the petrochemical industrial chain has been extended. The entire amount of production, usage, storage, transportation, import, and export of hazardous chemicals is also increasing, resulting in the rapid development of the hazardous chemical logistics industry. Despite the recent coronavirus pandemic, the market for hazardous chemicals and dangerous products logistics industry exceeds 2 trillion yuan. There were 13,000 hazardous chemical road transport enterprises in September 2020, employing 1.62 million workers and operating 575,000 transport vehicles nationwide. The transport volume is approximately 1.2 billion tons, accounting for 70% of the total transport volume [1]. With the rapid increase in the volume of hazardous chemicals transported by road in China, a high volume of hazardous chemicals appears on the urban road network as “mobile sources of danger” [2]. The occurrence of accidents, human factors, accidental causes, and disposal challenges are increased due to the mobility of hazardous chemicals through road transportation. When an accident occurs, it causes harm to drivers and escorts, severely threatens public safety and property along the transportation route of hazardous chemicals and causes irreversible pollution to the environment (soil and rivers). For example, in 2005, the liquid chlorine leak in the Huai’an section of the Beijing–Shanghai China Expressway caused 28 deaths, nearly 10,000 people were evacuated, 13.74 square kilometers of crops were affected, 15,000 livestock and poultry died, and the bright green wheat was contaminated into yellow [3]. In 2020, an explosion of liquefied petroleum gas leak on an Expressway in Zhejiang Province of China caused 20 deaths and 172 hospitalizations. In the same year, HCRTAs of different severity occurred in Nigeria, Mexico, India, and other countries, causing certain impacts [4]. Road transportation of hazardous chemicals is not only a challenge to public safety but an essential strategic and tactical decision-making challenge [5]. Therefore, scholars are increasingly concerned with the study of road transport safety of hazardous chemicals.

At present, scholars have studied the spatial and temporal characteristics of HCRTAs [6,7], types of hazardous chemicals [8,9], types and causes of accidents [10,11], and emergency disposal methods [12,13] via statistical analysis. Oggero et al. [14] studied 1932 accidents during the transport of hazardous chemicals by road and rail in 95 countries from the start of the 20th century to July 2004 and observed that 73.5% of hazardous chemical transportation accidents were caused by collisions. However, information on the number of persons evacuated in these hazardous chemical transportation accidents is lacking. Several scholars have developed a risk evaluation model for hazardous chemical road transportation to reduce the risk of HCRTAs [15,16,17,18,19,20,21,22], and simultaneously studied the emergency auxiliary decision-making system for hazardous chemical road transportation based on Internet technology [23,24,25,26,27,28,29,30]. Huang et al. developed a risk evaluation model as well as one for the road transport of hazardous chemicals based on GIS, suggesting factors such as exposure, vulnerability, and recoverability in the influenced region of hazardous chemical road transport [2]. However, there are relatively few studies on the public evacuation of HCRTAs.

Therefore, to explore the characteristics of HCRTAs in China and their emergency evacuation, this study developed a statistical analysis from 2012 to 2020. Then, information sources and analysis methods of HCRTAs are introduced. Based on the 2012–2020 data from China, five aspects of HCRTAs are analyzed in terms of time, space, trigger factors and accident types, distribution of injuries and fatalities, types of substances, and common HCRTAs are selected for analysis. The social risk of evacuation events in HCRTAs is then estimated and graded, including the following steps: (i) Determine the probability of several trigger factors (collision, tailgating, etc.) resulting in HCRTAs based on statistical data. (ii) Determine the probability of each trigger resulting in various event chains (leakage, leakage–fire, etc.) based on statistical data. (iii) Determine the evacuation trigger probability of each trigger factor resulting in various event chains and thus evacuation events based on statistical data. (iv) Estimate the evacuation scenario probability based on the accident trigger probability, event chain probability, and evacuation trigger probability. (v) Estimate the value of labor losses of the personnel involved in the emergency response. (vi) Estimate the cost of evacuation and relocation of personnel. (vii) Calculate the social cost of the evacuation event as a consequence of the evacuation event based on the value of the labor loss of emergency responders and the cost of evacuation and resettlement of personnel. (viii) Estimate the social risk of evacuation events according to the probability of evacuation scenarios and the implication of evacuation events. (xi) Classify evacuation events according to the social risk of evacuation events. After that, a structure for enhancing the emergency evacuation capacity of communities as well as road transportation of hazardous chemicals is proposed in five aspects: land planning and utilization, city construction, education promotion, information construction, and the layout of emergency resources. Then, discussion of the problems in conducting the study. Finally, conclusions are given.

## 2. Data and Methods

### 2.1. Data Source

The data for this study came from the China Chemical Accident Information Network (CCAIN; a platform for petrochemical accident statistics and data analysis) (Accident. NRCC, http://accident.-nrcc.com.cn, accessed on 1–31 December 2020). In addition, certain information about the accidents was obtained from local government websites or news reports. This study analyzed 2657 accidents involving hazardous chemical road transport in China from 1 January 2012 to 31 December 2020. Information regarding HCRTAs used in this study mainly includes time, location, types of chemicals involved, accident causes, trigger factors, accident types, casualties, and emergency information. The study explores all regions of the country (a few HCRTAs may be lost due to the continuous upgrading of the accident statistics system). Therefore, the statistical results can be used to analyze the characteristics and emergency development patterns of HCRTAs in China.

We filtered the database information year by year and month by month by selecting the year and month in the text search box. The search results include all hazardous chemical incidents for this period, but not all incidents meet the statistical criteria. The list of search results includes the title, date, and location of each event and a summary description of the event. Based on the event title and event summary, we determine if the event occurred during transportation and caused a spill, fire, explosion, etc., of the material being transported, and if so, the event meets the filtering criteria. If the information was incomplete or insufficient to determine whether the screening criteria were met, additional incident information was obtained from local government websites or news reports for further screening and data collection.

### 2.2. Statistical Methods

First, this study obtained valid information from accident reports and converted the data into a database in excel format. In addition, the statistical results are analyzed from the five aspects: time, space, trigger factors and accident types, distribution of injuries and fatalities, types of substances, and common HCRTAs. The evaluation indices of HCRTAs include accident occurrence, number of fatalities, mortality rate, accident trigger probability, event chain probability, and evacuation trigger probability. Accident frequency (L) refers to the number of HCRTAs in a certain period, a trigger factor (brake failure, rollover, etc.), or an event type (leakage, leakage–explosion, etc.).

The number of fatalities (*K*) is the number of fatalities in HCRTAs in a certain period. Mortality rate (*P*) refers to the number of deaths in each hazardous chemical accident, which can be expressed by the following equation:

(1) $$P{}_{q}=\frac{K{}_{q}}{L{}_{q}}$$

where q refers to the year (or month) of the accident; $L_{q}$ refers to the accident frequency of HCRTAs in the *q* period; $K{}_{q}$ refers to the number of deaths in HCRTAs in the *q* period; $P_{q}$ refers to the mortality rate of HCRTAs in the *q* period.

Accident occurrence rate (*I*) refers to the proportion of a certain type of accident (trigger factor or event chain type) in the total number of road transportation accidents (*W*) involving hazardous chemicals, which can be expressed by the following equation:

(2) $$I_{i}=\frac{L_{i}}{W}$$

(3) $$I_{j}=\frac{L_{j}}{W}$$

(4) $$H_{ij}=\frac{L_{ij}}{L_{i}}$$

where, i refers to accident trigger factors (brake failure, rollover, etc.); $L_{i}$ indicates the number of HCRTAs caused by trigger factor *i*; $I_{i}$ accident trigger probability refers to the probability of HCRTAs caused by trigger factor *i*; *W* refers to the total number of HCRTAs counted in this paper. j indicates the type of event chain (leakage, leakage–fire); $L_{j}$ represents the number of HCRTAs with event chain type *j*; $I_{j}$ the accident rate of event chain type *j*. $L_{ij}$ refers to the number of HCRTAs in event chain *j* caused by a trigger *i*; $H_{ij}$ event chain probability refers to the probability of HCRTAs of an event chain type caused by a trigger factor.

The number of evacuation events (*Y*) is the number of accidents in which evacuation information is provided (alert area, evacuation area, number of evacuees, evacuation time, emergency disposal time, etc.) in the total number of HCRTAs (*W*) collected in this study. $E_{ij}$ evacuation trigger probability refers to the evacuation event probability of event chain type *j* caused by trigger factor *i*, which can be expressed by the following equation:

(5) $$E_{ij}=\frac{Y_{ij}}{Y_{i}}$$

where $Y_{ij}$ represents the number of evacuation events of event chain *j* caused by trigger *i*; $Y_{i}$ represents the number of evacuation events caused by trigger *i*.

## 3. Characteristics of HCRTAs

### 3.1. Characteristics of Time Distribution

**Figure 1:**
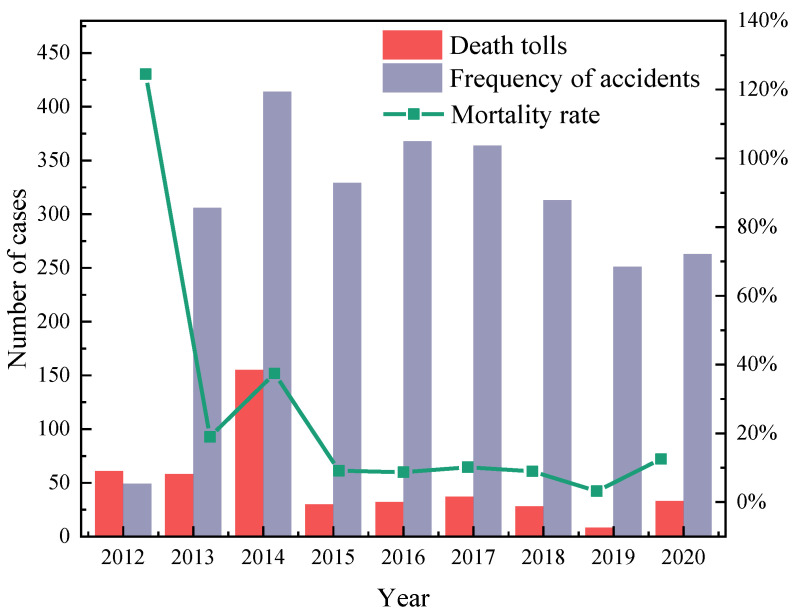
Statistics on the frequency, number of deaths, and mortality rate of HCRTAs from 2012 to 2020.

Figure 1 shows HCRTAs from 2012 to 2020. Accidents increased in occurrence progressively from 2012 to 2014. In 2014, the occurrence of accidents was at its highest, at 414. This could be due to China’s industrial transformation during the 12th five-year plan period, the rapid development of the chemical industry, and the overall yearly increasing trend of accident distribution. In 2015, the rate of accidents dropped drastically compared with that of 2014, at 329. The explanation may be that after the occurrence of two major accidents in the transportation of hazardous chemicals in China in 2014, the government paid more attention to the safety challenges in this sector, and the supervision was strict. According to the data, significant measures have had some success. In 2016, there was a slight increase in the number of accidents compared with 2015. Yearly, the number of accident occurrences decreased from 368 to 251, probably because of the implementation of the revised regulations of risky goods by road transport in 2016 [7]. In 2020, the number of accidents slightly increased. Beginning 1 January 2020, the safety management procedures for the transport of risky goods by road were applied. This demonstrates that when the laws and regulations are first implemented, the occurrence of accidents may increase slightly. This is consistent with the safe Kuznets curve [31].

In 2012, the highest accident mortality rate was 120%. This is mainly due to the “8.26” major road traffic accident in Yan’an, Shaanxi Province on the Baomao Expressway, and the oil tanker explosion in Guangzhou, both of which resulted in 36 and 20 deaths, respectively. The accident mortality rate was also relatively high in 2014. The reason is the occurrence of the “7.19” major accident in the Shaoyang region of Hunan Province of the Shanghai Kunming Expressway and the “3.1” accident in the Shanxi Jincheng Yanhou tunnel of the Shanxi Jinan Expressway, with a death toll of 54 and 40, respectively. The accident mortality remained within 10% from 2015 to 2019. One possible cause of this is that after 2014 the relevant departments increased the supervision of road transportation of hazardous chemicals. The main reason for the increase in accident mortality in 2020 is the major explosion accident of a “6.13” liquefied petroleum gas (LPG) transportation tank truck in the Wenling region of the Shen Hai Expressway. As a result, preventing the occurrence of major casualties is a significant aspect of reducing the risk of HCRTAs.

**Figure 2:**
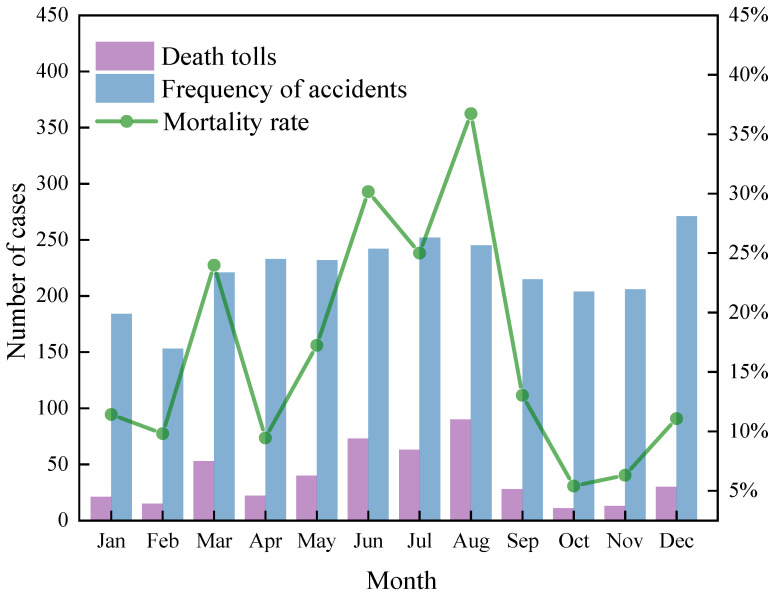
Statistics on the frequency, number of deaths, and mortality rate of HCRTAs for every month.

Figure 2 shows the monthly distribution of HCRTAs from 2012 to 2020. The number of accidents from January to February is relatively low, with <190 accidents per month. During the Chinese Spring Festival, people go home to celebrate the Spring Festival, which results in a reduction in the trading and transportation of hazardous chemicals. During this period, the authorities in charge of hazardous chemicals toughened their regulations. In trading traffic growth from March to October, the number of accidents increased significantly, with the number of accidents exceeding 240 per month in June, July, and August. HCRTAs are most common during the summer months of June–August. Because of the hot weather and high temperature in summer, hazardous chemical vehicles are involved in automobile and tire fire accidents during transportation. The rise in temperature and pressure will result in the volatilization of hazardous chemicals, transport container valves to fail, and container rupture, among others. November and December saw an increase in the number of accidents, with December reaching 271 cases, which was higher than those of the summer months of July and August. This is due to an increase in the demand for gas transportation and fuel-based hazardous materials as the fall and winter seasons are approaching. Furthermore, due to the winter snow days in December, there will be more foggy days, low visibility, and an increased risk of traffic accidents.

From June to August, the mortality rate of HCRTAs is high. The highest mortality rate was in August at 36.73%. Although the number of accidents was the highest in December, the mortality rate was lower at 11.07% because the higher temperature in summer makes it easier for hazardous chemicals to react during transportation, resulting in secondary accidents.

**Figure 3:**
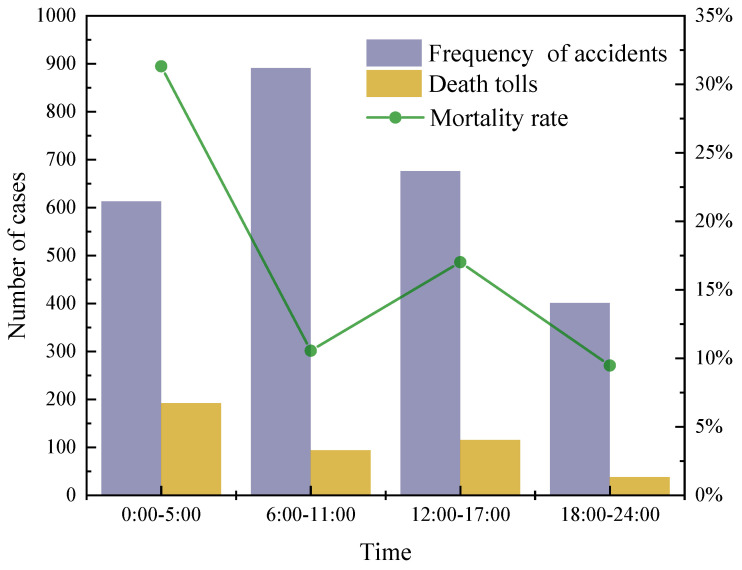
Accident frequency, death toll, and mortality rate of HCRTAs at different times.

As shown in Figure 3, among the 2657 accidents recorded, the occurrence time of 2581 accidents was obtained. In the statistical process, a day is divided into four intervals at 6-h intervals, which are separated for the morning (06:00–11:00) (time division description: 06:00–11:00 implies 06:00–11:59, 12:00–17:00, 12:00–17:59, and so on), afternoon (12:00–17:00), the first midnight (18:00–23:00), and the second midnight (0:00–05:00). There were 891 accidents in the morning, 767 in the afternoon, 401 in the first half of the night, and 601 in the second half of the night from the time of the accident. The occurrence of accidents is highest in the morning. Between 06:00 and 09:00 a.m., medium and long-distance transport vehicles are out, with many heavy trucks on the highway at that time, resulting in a high rate of vehicles in the traffic flow, which is one of the reasons for the high incidence of HCRTAs during that time. However, in the second half of the night, the mortality rate was higher, reaching 31.32%. Driver tiredness deepens, and the ability to cope with accidental emergencies decreases due to the lowest level of human arousal in the second half of the night (0:00–05:00) [32]. In addition, due to the low visibility at night, drivers tend to believe that there is no other vehicle on the road during this time and speed up, resulting in accidents [6].

### 3.2. Characteristics of Spatial Distribution

**Figure 4:**
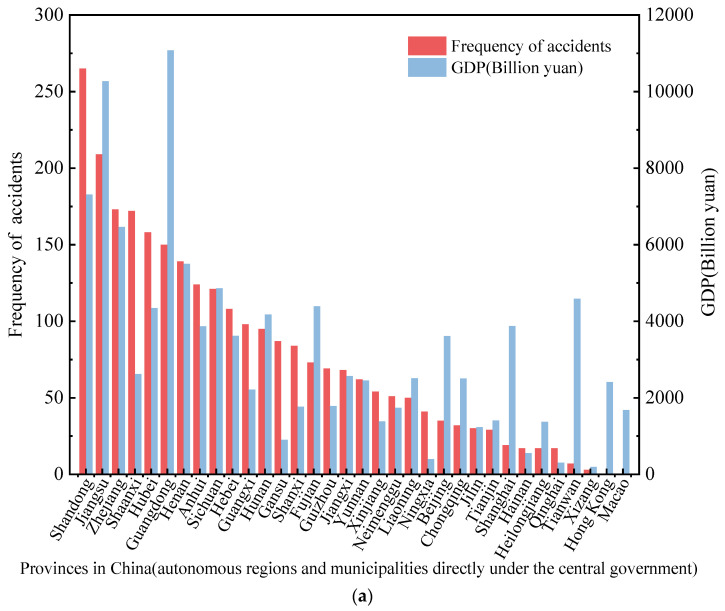

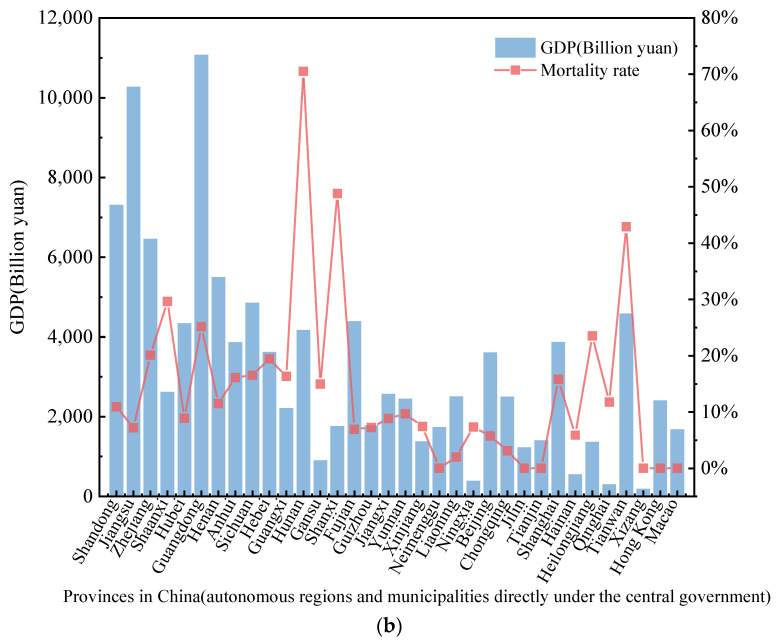
(**a**). Frequency of HCRTAs in all provinces (autonomous regions and municipalities directly under the central government) and annual GDP in China from 2012 to 2020. (**b**). The mortality rate of HCRTAs and annual GDP of all provinces (autonomous regions and municipalities directly under the central government) in China from 2012 to 2020.

We analyzed the road transportation accidents and mortality rate due to hazardous chemicals in provinces, municipalities, regions, and road types from 2012 to 2020. Figure 4a shows the spatial distribution of accident occurrence and annual GDP (2020 GDP) by province. The highest occurrence of hazardous chemical transportation accidents is in Shandong Province and Jiangsu Province, 265 and 209, respectively. The accident occurrence in Shandong Province is higher than that of Jiangsu Province, which is different from the leakage of hazardous chemicals [9,33]. Zhejiang, Shaanxi, Henan, and Guangdong, with more than 150 accidents in each of the four provinces. The occurrence of accidents in Hainan Province, Heilongjiang Province, Qinghai Province, Tibet Autonomous Region, Taiwan Province, and two special administrative regions is less than 20. Since most of China’s chemical enterprises are located along the coast, the frequency of accidents in coastal regions is high. According to the 2020 GDP results from China’s National Bureau of Statistics for each province, seven provinces, including Guangdong, Jiangsu, Shandong, Zhejiang, Henan, Sichuan, and Fujian, have high total GDP and relatively developed industries and economies. The development of regional economies and special industries makes the cross-provincial circulation of hazardous chemicals common, one of the reasons for the increase in accidents.

However, the high or low mortality rate of HCRTAs in each province is not positively connected with the occurrence of accidents, as shown in Figure 4b. The provinces with the highest mortality rate were Hunan and Shanxi, with 70.52% and 48.8%, respectively, followed by Taiwan, Shaanxi, Guangdong, and Heilongjiang. Provinces such as Hunan, Shanxi, Taiwan, and Heilongjiang do not have high GDP and accident occurrence (in terms of ranking), but their mortality rate is relatively high. This demonstrates that in some provinces where the chemical industry is not developed, the occurrence of accidents is low. It may be that the roads in the above areas are in mountainous areas, so no effective measures can be taken to control the consequences after the accident. Shaanxi Province is ranked 15th in GDP (2020), and its accident occurrence and mortality rate are high. The local government should strengthen the supervision of the transportation of hazardous chemicals, strengthen the optimization of transportation routes, and improve the emergency disposal capacity.

As shown in Figure 4a,b, Shandong and Jiangsu Provinces have the highest accident occurrence rate, and the mortality rate is 10.94% and 7.17%, respectively. Hunan Province and Shanxi Province, which have low accident occurrences, have the highest mortality rates. Guangdong and Shaanxi provinces rank high in the frequency of accidents and mortality rate. At present, there are still some shortcomings in the transport supervision and management of dangerous chemicals, for example, some monitoring networks are now operating independently, and can only monitor dangerous goods transport vehicles in the region and within the enterprise. Some hazardous chemical transportation routes are not set up for all-around monitoring. Therefore, the emergency response capability of professional emergency responders and local people in underdeveloped areas of the hazardous chemical industry should be enhanced. The government should intervene before a major accident occurs through effective emergency management measures such as dynamic monitoring of hazardous chemicals, and early warning of abnormal situations.

**Figure 5:**
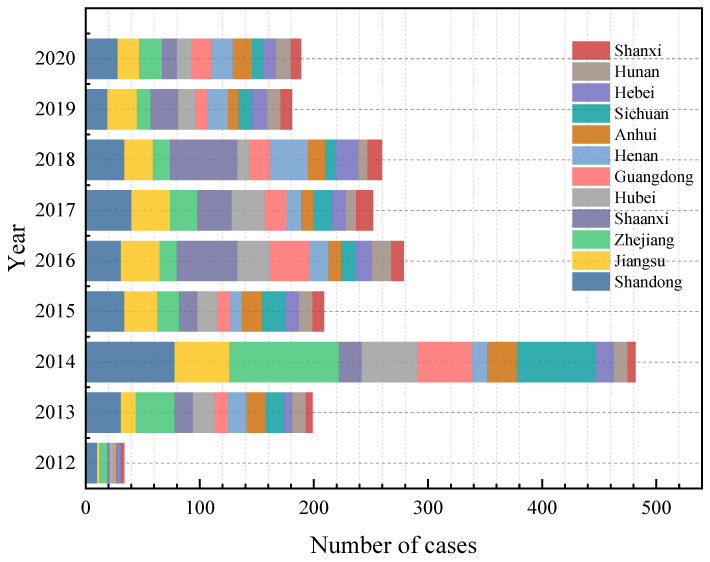
Number of HCRTAs by province from 2012 to 2020.

Figure 5 shows the results of a statistical analysis of the provinces with high accident frequency or mortality. From 2012 to 2020, the frequency of accidents in eight provinces, namely, Shandong, Jiangsu, Zhejiang, Hubei, Guangdong, Anhui, Sichuan, and Gansu, reached the highest in 2014, and the frequency of accidents in the year is more in Zhejiang Province than in Shandong Province. In 2018, three provinces, namely Shaanxi, Henan, and Hebei, had the highest frequency of accidents. Compared with those of 2014 and others, the difference between the frequency of accidents in the 12 provinces recorded is gradually narrowing. This is related to the booming development of China’s chemical industry. At the early stage of development, China’s regulatory measures for hazardous chemicals were far from perfect, leading to an increasing number of accidents. After 2014, China began to strengthen the regulation of hazardous chemical transportation, and enterprises learned about major HCRTAs, paying more attention to the safety of hazardous chemical road transportation, and the number of HCRTAs decreased in most provinces.

**Figure 6:**
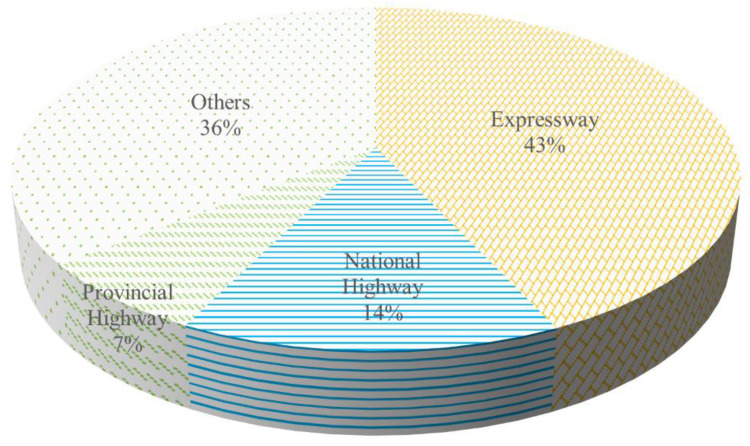
The proportion of accidents involving road transportation of hazardous chemicals on different road types.

In the accident road type statistics, due to the limited accident information collected, this study divides the road types into four categories: expressway (including urban ring roads), national highway, provincial highway, and others (roads other than the above three and those for which information is not available). Figure 6 shows the accident distribution. Expressway accidents reported 43%, national roads reported 14%, provincial roads reported 7%, and others reported 36%. This may be because the expressway is the major means of transporting hazardous chemicals across provinces, and the total number of transportations is high; thus, the number of accidents is the highest. The road conditions on national and provincial highways are poor compared with the expressway, which results in accidents.

### 3.3. Trigger Factors and Accident Types

#### 3.3.1. Trigger Factors

**Figure 7:**
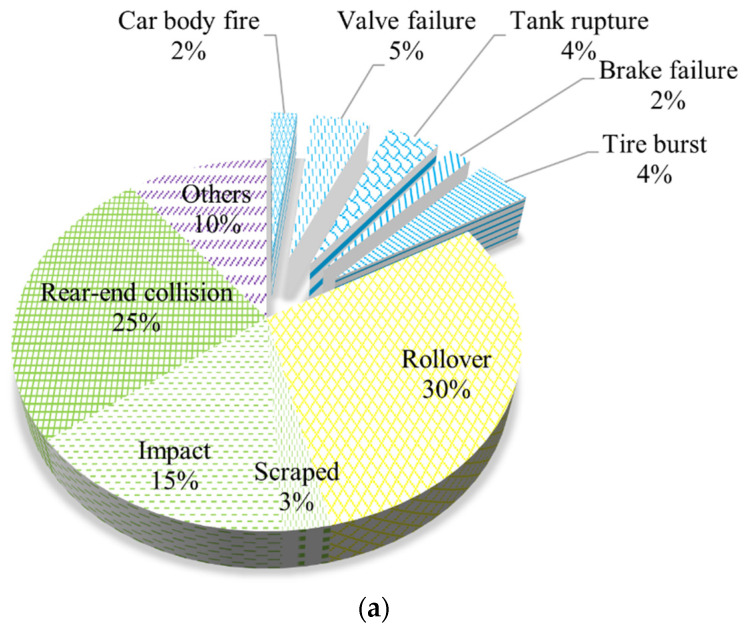

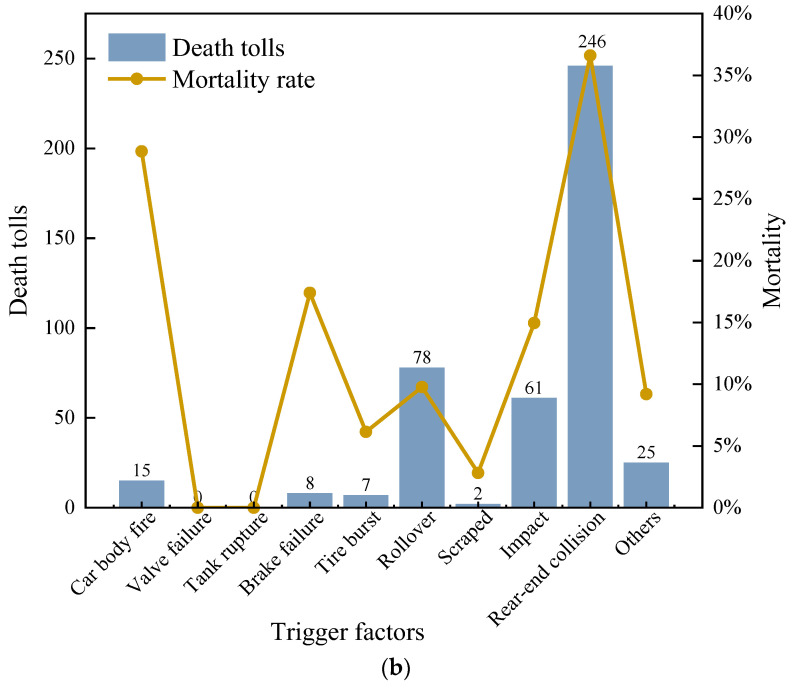
(**a**). The frequency of HCRTAs is caused by different trigger factors. (**b**). Death tolls and mortality rate of HCRTAs caused by different trigger factors.

The trigger factors of hazardous chemicals in road transportation accidents can be divided into ten categories: vehicle fire, valve failure, tank rupture, brake failure, tire burst, rollover, cut, impact, rear-end, and others (information on trigger factors is not available). The statistical results are shown in Figure 7a. Rollover and rear-end accidents reported a higher occurrence of accidents, 30% and 25%, respectively. The reported rates of a car fire, valve failure, tank rupture, brake failure, and tire blowout are 2%, 5%, 4%, 2%, and 4%, respectively. The reasons for this are as follows: (1) road, weather, and other unavoidable factors. (2) Drivers are tired and lack safety awareness. (3) The transport vehicle is not regularly serviced, resulting in valve gaskets or oil pipeline aging, which leads to valve failure. (4) As the vehicle line fails, a short circuit occurs, causing the vehicle body to heat up. In addition, the temperature is high in summer, and the vehicle speed is extreme, making it easier for the tire to heat up. (5) The transport vehicle fails to meet the qualification for hazardous chemicals transportation, or the transportation qualification has expired, and the transport vehicle is illegally overloaded.

Figure 7b shows the number of deaths and mortality rate caused by ten types of trigger factors. Rear-end collision has the highest number of deaths and mortality rate. Although the total number of deaths caused by car body fires is low, the average number of deaths per accident is high. Rollover is the biggest factor that triggers accidents, but its mortality rate is only 10% due to: (1) Although the number of accidents triggered by rollover is high, their scope is limited to the accident vehicles and the surrounding regions. Accidents triggered by rear-end collision affects several vehicles and their surrounding regions, which have a higher impact. (2) When compared with other trigger factors, accidents triggered by a car body fire is likely to result in secondary injury to persons. As a result, individuals should be aware of HCRTAs caused by rear-end collisions and car body fires.

In addition, enterprises should increase safety training for drivers, improve drivers’ safety awareness and skills, and prevent tired drivers from driving. Vehicles transporting hazardous chemicals should be inspected before driving to ensure there are no challenges with aging lines and tires. Vehicle maintenance and qualification inspection should be performed regularly to ensure vehicle safety to effectively prevent HCRTAs.

#### 3.3.2. Types of Event Chains Caused by Different Triggers

**Figure 8:**
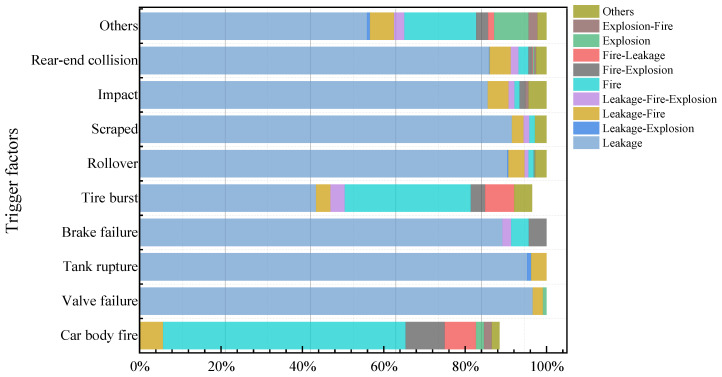
Probability of different trigger factors leading to different event chain types.

Figure 8 shows the statistical results of several event chains generated by different trigger factors leading to HCRTAs from 2012 to 2020. The event chain is divided into ten categories: leakage, leakage–explosion, leakage–fire, leakage–fire–explosion, fire, fire–explosion, fire–leakage, explosion, explosion–fire, and others (unable to obtain event chain information). There is a 59.62% chance of fire caused by body fire and a 7.69% chance of fire–leakage. Valve failure has a 96.64% chance of causing leakage and a 2.25% chance of causing leakage–fire. The possibility of an explosion caused by valve failure is quite low. A puncture on the tank body results in a chain of events dominated by leakage. Since brake failure will result in a violent collision, the leakage event chain would lead to leakage–fire–explosion, fire, and fire–explosion event chains. The tire burst has a 30.97% chance of causing a fire event chain, which is the most likely to start a fire event chain in addition to the car body fire. Most event chains are initiated by rear-end collisions. The accident consequences caused by rear-end collisions are affected by many factors: vehicle speed, road environment, and road traffic flow.

#### 3.3.3. Event Chain Type

**Figure 9:**
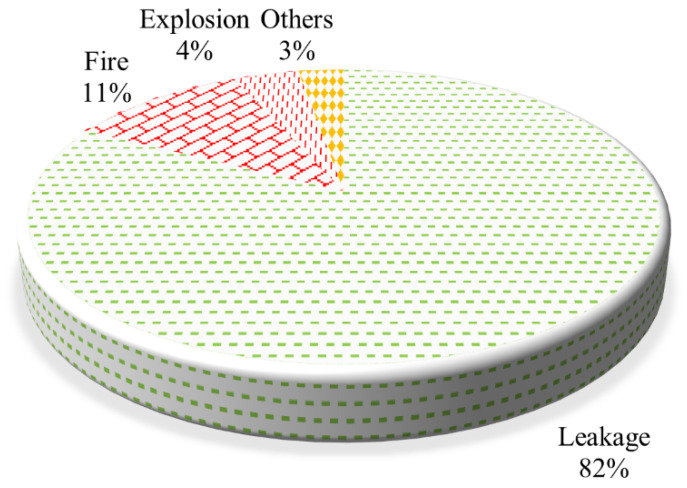
Statistics on types of HCRTAs from 2012 to 2020.

Figure 9 shows the types of HCRTAs from 2012 to 2020. Leakage accidents accounted for 82%, fire for 11%, explosion for 4%, and others for 3% (there are accidents, but there is no leakage, fire, or explosion in the accident). The final manifestation of the accident is reported as accident type. For example, the explosion accident may be a leakage–fire–explosion event chain or a leakage–explosion event chain. The fire accident may be a leakage–fire event chain, or a fire event chain directly caused by vehicle body fire.

Table 1 shows the evolution process and strength of several event chains. There were 2176 leakage occurrences in all accidents, accounting for 81.90% of the total. A total of 124 leakage–fire occurrences accounted for 4.67% of all accidents, 5 leakage–explosion event chains accounted for 0.91% of the total, and 44 leakage–fire–explosion accidents accounted for 1.66%. This indicates that 1 out of every 17.5 leakage incidents will result in a fire incident, and every 2.9 leakage–fire incidents will result in an explosion. There were 148 accidents in the fire incident chain, which accounted for 5.57% of the total number of accidents, 17 fire–leakage occurrences, accounting for 0.64%, and 31 fire–explosion occurrences, accounting for 1.17%. Thus, for every 4.77 accidents where the initial event is a fire, there is an explosion.

Among the ten types of event chains, the leakage–fire–explosion event chain has the highest mortality rate at 427%. On average, at least four persons die because of the leakage–fire–explosion event chain. The mortality rate of the explosion–fire event chain is 107%. In the explosion–fire event chain, an average of at least one person dies. The mortality rate of the explosion chain is 75%. For each explosion chain accident, an average of one person dies. The mortality of the fire–explosion chain is 68%. On average, about one person dies for every accident in the fire–explosion chain. It shows that the chain of events containing the explosion-type event chain results in severe consequences. The mortality rate due to leakage–explosion is 0. The accident occurrence of the leakage–explosion chain is only 2 in the statistical process, which does not cause death. Due to the physical explosion that occurred in the process of accident handling, the severity of the accident is relatively low. Fire–leakage also did not cause death, mainly because when the car body caught fire, due to timely measures to put out the fire, material leakage was prevented, and the degree of injury was relatively low.

### 3.4. Distribution of Injuries and Deaths

**Figure 10:**
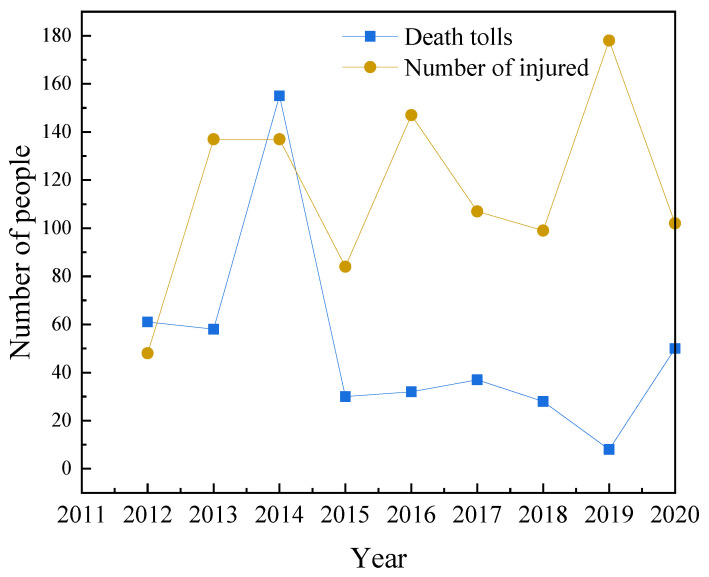
Number of fatalities and injuries of HCRTAs from 2012 to 2020.

In the 2657 accidents, 459 people died and 1039 were injured. In 2012 and 2014, there were more deaths, as seen in Figure 10. This was due to the particularly serious road transport accidents involving hazardous chemicals in 2012 and 2014, which caused many deaths. The changing trend in the number of injuries from 2014 to 2018 is like that in the number of deaths. In 2019, the number of deaths was the lowest, but the number of injuries was the highest, reaching 178. This is because a tanker transporting liquid ammonia collided with a truck, causing liquid ammonia leakage, and triggering vomiting and dizziness symptoms in 130 persons in varying degrees along the road. In 2020, the number of injuries caused by HCRTAs declined slightly, but the number of fatalities increased significantly.

The accident fatality grade is shown in Table 2, 93.6% of accidents did not cause death, 5.61% of accidents caused one to two deaths (most of them are drivers and escorts), and 0.79% of accidents caused three or more deaths.

The accident injury grade is shown in Table 3. Of the 2657 accidents, 84.42% resulted in no injuries, 13.21% had 1–2 injuries, 1.88% resulted in 3–9 injuries, and 0.49% resulted in more than 10 injuries. When the number of injured persons is one to two, the majority are caused by vehicles. Accidents with many injuries (more than 10 people) are caused by fire and explosion.

### 3.5. Types of Materials

According to China’s classification and code of dangerous goods [34], dangerous goods are classified into nine categories. When a chemical has more than one of these properties at the same time, the standard specifies the status of the hazards in Section 5.3, (1) explosives, (2) gases, (3) flammable liquids, (4) flammable solids (substances that are prone to spontaneous combustion, substances which, in contact with water, emit flammable gases), (5) oxidizing substances and organic peroxides, (6) toxic and infectious substances, (7) radioactive substances, (8) corrosive substances, and (9) miscellaneous dangerous substances and articles, including environmentally hazardous substances. In this study, hazardous chemicals are classified according to the characteristics that rank highest in terms of hazard.

**Figure 11:**
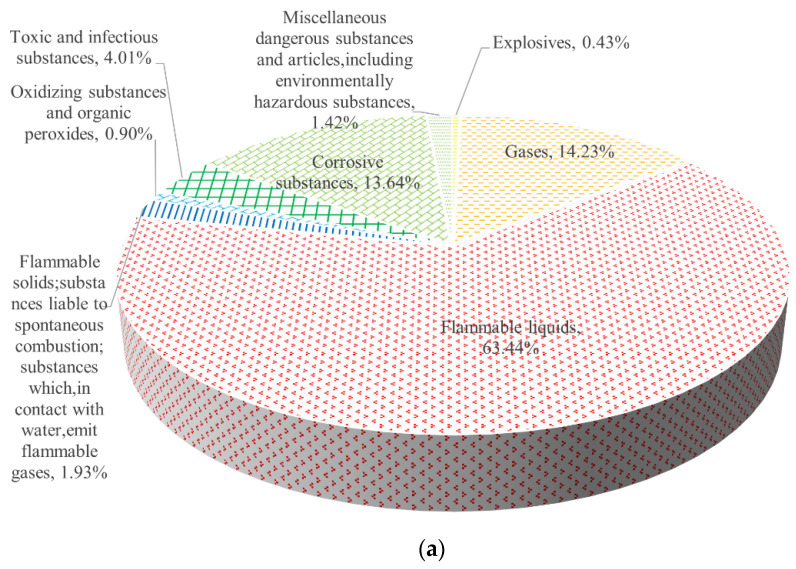

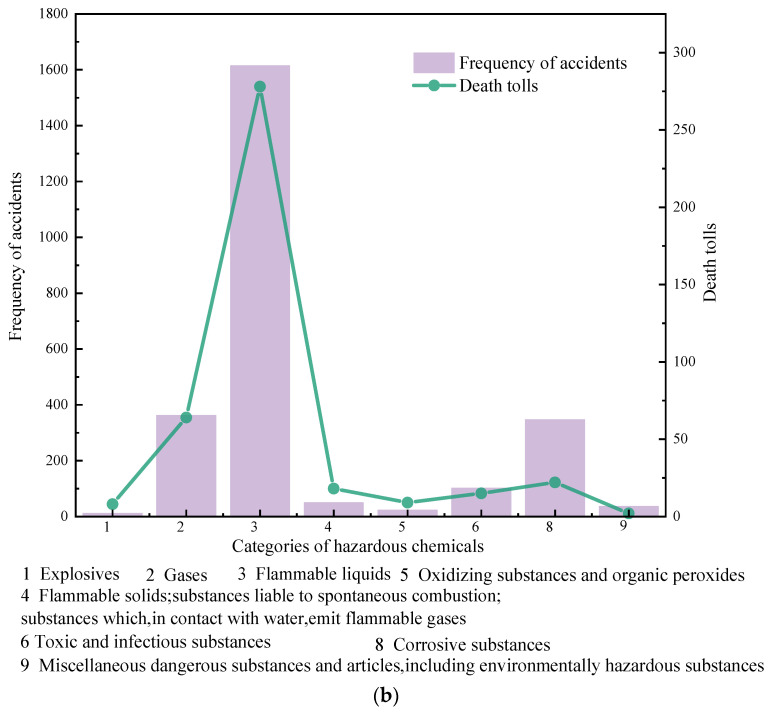
(**a**). Frequency of HCRTAs of different hazardous chemical types. (**b**). Frequency of HCRTAs of different hazardous chemical types.

As seen in Figure 11a, flammable liquids (typical materials in the statistical process: diesel, gasoline, methanol, etc. Methanol is flammable, its vapor and air can form explosive mixtures, and it has a narcotic effect on the human central nervous system) accounted for 63.44% of all accident materials. Most of the transport materials for gases are natural gas and liquefied gas, and a low amount of carbon dioxide and other compressed non-flammable gases, so flammable substances account for at least 70%. Corrosive substances (typical materials in the statistical process: sulfuric acid, nitric acid, hydrochloric acid) accounted for 13.85%. Sulfuric acid and nitric acid accidents are the most common, but they cause fewer casualties. Chemicals such as bromine and phosphorus trioxide corrode and volatilize after leaking, producing large toxic gases, which mostly cause only mild or moderate poisoning, but cause greater social impact. A total of 4.01% of toxic and infectious substances (typical materials in the statistical process: methamidophos, phenol, etc. Phenol can be ignited by high heat in an open fire and has a strong corrosive effect on the skin and mucous membranes can inhibit the central nervous system and damage liver and kidney function), and 0.90% of oxidizing substances (typical materials in the statistical process: hydrogen peroxide).

Figure 11b shows that flammable liquids, gases (typical materials in the statistical process: natural gas, liquefied petroleum gas, liquid ammonia, liquid chlorine, etc.), and toxic and infectious substances are the main media causing casualties. Such materials easily cause significant social impact, such as leakage of flammable liquid and gas causes pool fires, gas clouds, explosions, and other serious consequences. These accidents have a higher risk effect, fast diffusion speed, and are widespread. In addition, the risk of exposed persons in the road section is high, which easily leads to a high number of casualties. Gases of typical substances such as liquefied gas, ethylene oxide, and hydrogen have caused large numbers of residents to be evacuated. Accidents of contamination of water bodies by leaking toxic and infectious substances have seriously damaged the water environment, caused social panic, and in some cases caused poisoning of downstream residents. Therefore, for toxic and infectious substances, special attention needs to be paid to the selection of driving routes should avoid important water sources such as reservoirs, lakes, and rivers as far as possible. Flammable solids (typical materials in the statistical process: sulfur, yellow phosphorus, calcium carbide, etc. Calcium carbide can produce highly flammable acetylene gas when it meets water, which can also cause human skin burns) also caused 18 deaths. The main reason is that the transport medium accidentally encountered water in the transport process and the occurrence of natural, transport personnel about the nature of such substances do not understand thoroughly. Therefore, the professional skills training of practitioners should be improved to master the safety technology of the risky transportation of hazardous chemicals and regulate the operation to avoid accidents.

### 3.6. Typical Accidents

To propose strategies for reducing the risk of HCRTAs, we examined six accidents that resulted in a high number of casualties and severe societal implications for analysis. As shown in Table 4, all six accidents occurred on the expressway, four accidents were triggered by rear-end collision, and five accidents were caused by a leakage–fire–explosion event chain. Secondary accidents such as fire and explosion are significant causes of death during HCRTAs. Most of the fires and explosions occur due to rear-end collisions or overturning and other trigger factors caused by high external forces acting on the tank or vehicle, resulting in leaks after a delay in plugging the leak (external forces might puncture the tank or short-circuit the body wire, which generates sparks, leading to the burning and explosion of the leaking material). In addition, due to the difficult regions of the accident site during transportation, it is challenging to control ignition sources such as sparks. Therefore, we must implement the “persons,” “vehicles,” and “tanks” safety improvement project to significantly improve the quality of personnel, vehicle safety strategies, and tank safety technology to effectively eliminate major safety risks in the transportation of hazardous chemicals.

Table 4 shows that the primary causes of HCRTAs and several fatalities are as follows: (1) Road transport enterprises transporting hazardous chemicals have illegal refitting of vehicle tanks, overloading, speeding, tired driving, and other behaviors. (2) The population density near the accident site is relatively high due to several reasons. (3) The emergency evacuation is insufficient, and the emergency rescue process lacks sciences. The drivers and escorts engaged in the transportation of hazardous chemicals have insufficient emergency awareness and low emergency evacuation skills. There is a lack of transportation information exchange between various regulatory departments in all provinces, and there is a lack of professional hazardous chemical emergency rescue management networks and emergency rescue management mechanisms. The accident resulted in 20 deaths, 175 hospitalized (including 24 serious injuries), and direct economic losses of 94,778,150 yuan.

**Figure 12:**
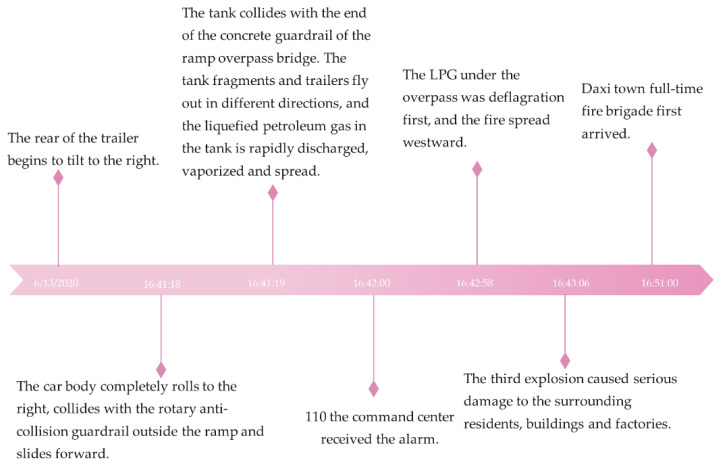
Timeline of the “6·13” Wenling tank car explosion accident.

The “6.13” Wenling tanker explosion is a major production safety responsibility accident. On 13 June 2020, at 16:41:00, a major explosion of an LPG tanker occurred on the Wenzhou-bound Wailing West exit ramp of the Shen Hai Expressway, Taizhou Wailing City. The accident timeline is demonstrated in Figure 12. The accident occurred while the vehicle drove from the road section with a speed limit of 60 km/h to the curve section with a speed limit of 30 km/h. The vehicle was overturned due to late deceleration, resulting in a rollover of the vehicle, and a violent impact between the front of the tank and the end of the concrete guardrail of the overpass bridge. The tank was torn and disintegrated under the impact of the pressure. The rapidly released, vaporized, and diffused LPG was deflagrated by sparks from passing motor vehicles and caused a steam cloud explosion.

The front of the tanker was thrown approximately 100 m away by the first physical explosion and landed on the lawn below the side of the expressway. The horizontal storage tank containing LPG failed and broke, flying approximately 360 m away from the accident site. The 26t liquefied petroleum gas was instantly dumped on the lawn of the site, which vaporized and diffused, causing a vapor cloud over the residential area in the northwest. The LPG vapor cloud onsite was ignited at 16:42:58, the second explosion occurred, and the fire spread rapidly. At 16:43:06, the LPG vapor cloud onsite was ignited again, leading to the third explosion with rapid-fire propagation. The third explosion caused serious damage to the surrounding residents, buildings, and factories [35,36].

The distance from the edge of the expressway to Liangshan village is 27 m, the distance to the village houses is 36 m, and the nearest distance to Liangshan industrial zone is 65 m [37] according to the distance measured by the Baidu map. According to Article 11 of the regulations on expressway safety protection [38]: if it belongs to an expressway, the distance from the edge of the expressway land to the expressway building control region should not be less than 30 m. Therefore, there should be no other buildings within 30 m of the edge of the expressway. However, the range of the expressway building control area meets the requirements. According to Article 14 of the regulations on expressway safety protection: the distance between the newly built villages and towns, development zones, schools and commodities distribution centers, large commercial outlets, farmers’ markets, other public places, and the edge of the boundary expressway construction control region should not be <50 m. Therefore, the distance between Liangshan village and the expressway does not meet the requirements. The distance between the road and the industrial area is not specified in the road safety protection regulations (it is not explored whether villages and industrial regions are established first or expressways). However, according to the calculation of Hou et al. [36], the death radius of the explosion is 40 m, the serious injury radius is 90 m, and the minor injury radius is 176 m. Therefore, the distance between the village, industrial zone, and the expressway is in the region of significant injuries even when they implement road safety protection regulations, and the safety of persons is challenging to be guaranteed.

As shown in Figure 12, it takes only 2 s for the vehicle’s body to accelerate due to turning at the first physical explosion, which is 110 s away from the third explosion with significant consequences, that is, less than 2 min. In Table 4, in the “7.19” particularly severe accident in the Hunan Shaoyang region of the Shanghai Kunming Expressway, it took only 7 s for the rear-end fire between the truck and bus (spark generated by a short circuit during vehicle impact and fire at the time) to engulf the whole bus. The Bao Mao Expressway in Shaanxi Yan’an’ “8.26” is a major road traffic accident, and it took a relatively short time from the impact of the explosion. On 1 June 2015, at 21:32, the capsizing accident of the Oriental Star passenger ship occurred in China. It took 7 min for the passenger ship to retreat to the right rear due to the strong wind, which capsized the passenger ship. In the Hurricane Katrina event in the United States in August 2005, the National Hurricane Center released hurricane information on 23 August 2005. The hurricane approached the Gulf of Mexico on 25 August 2005, and made landfall in New Orleans on 29 August 2005 [39]. From the time hurricane information was released, it took six days for hurricane Katrine to land. The time from accident precursor to accident occurrence is different for several emergencies. The time of HCRTAs from accident omen to accident occurrence is short. HCRTAs progress extremely fast, increasing the difficulty of human intervention.

As a result, it is worth considering if it is necessary to invest a lot of labor and material resources to develop the early warning system. Therefore, to reduce the risk of HCRTAs, the following strategies can be taken: (1) comprehensively improve the safety skills of business employees. Furthermore, effectively strengthen the safety training, education, and management of employees and strive to enhance the legal awareness of enterprise employees as well as their safety awareness and safety skills. (2) Improve the fundamental safety of vehicles and tanks. The relevant state departments should further improve the technical standards and specifications for tank-type hazardous chemical transport vehicles according to the needs of road traffic safety management. Improve the strength of the rear-lower protection devices of vehicles for the transport of hazardous chemicals. Optimize the connection methods of vehicle tank valves and other devices. Improve the passive safety of tank-type hazardous chemical transport vehicles. (3) Improve the expressway standard system. Relevant governmental bureaus should further improve the expressway technical standards system, increase the acceleration and deceleration lane length of expressway service region exits, and set refined standards for inflow regions, physical isolation facilities, and safety distance, among others. (4) Strengthen emergency awareness and emergency response capability of the public in places where hazardous chemical transportation accidents often occur.

## 4. Classification of Evacuation Events Caused by HCRTAs

Generally, risk can be defined as the multiplication of the probability of an event and its consequences [40]. The International Strategy for Disaster Reduction [41] defines risk as the occurrence probability of damage resulting from the interaction between hazards and vulnerabilities. Social risk considers the vulnerability of the population and represents the product of the probability of potentially dangerous events among the exposed population [42]. Although HCRTAs could result in several major implications (such as damage to the ecological environment and economic losses), almost all studies in this field are related to the death of persons. It is generally common that the major implications are directly proportional to the population size of the incident region. The possibility of an accident depends on the type of substance and road that it carries [43]. In this study, the chances of triggering personnel evacuation due to adverse events are related to the social cost of the process of evacuation. The modeling idea of the algorithm is that the social risk of HCRTAs is affected by the probability of evacuation scenarios and the social cost of evacuation. The calculation equation is shown in (6):

(6) $$R_{ij}=F_{ij}\times C$$

where $R_{ij}$ refers to the social risk of evacuation caused by the event chain, the trigger factor $F_{ij}$ represents the probability of evacuation scenario, and C represents the social cost of evacuation.

### 4.1. Evacuation Scenario Probability

In the 2657 HCRTAs, 148 accidents involved evacuation information (evacuation events; the statistical process includes any one of the following information: alert range, evacuation range, number of evacuees, evacuation time, and emergency disposal time). The emergency information of certain accidents may be biased due to incomplete access to accident information data.

The probability of evacuation scenario refers to the probability that a certain event chain (leakage–fire–explosion) occurs due to a trigger factor (for example, rear-end collision) during the transportation of hazardous chemicals, which results in an evacuation event, expressed as $F_{ij}$. It can be expressed by the following equation:

(7) $$F_{ij}=I_{i}\times H_{ij}\times E_{ij}$$

where the subscript *i* indicates the accident trigger factors. $I_{i}$ represents the accident trigger probability. The subscript *j* indicates the event chain. $H_{ij}$ represents the probability of occurrence of event chain *j* due to trigger *i*. $E_{ij}$ represents the probability of an evacuation event caused by event chain *j* caused by trigger *i*.

#### 4.1.1. Accident Trigger Probability

Accident trigger probability refers to the probability of vehicle body fire, valve failure, tank rupture, brake failure, tire burst, rollover, scratch, impact, rear-end collision, and other factors (except the above 9 factors) during the transportation of hazardous chemicals, expressed as $I_{i}$. According to the statistical results in Section 3.3.1, the probability values of each trigger can be obtained, as demonstrated in Table S1 in the Supplementary material. The cases where the trigger is “others” are not studied here.

#### 4.1.2. Event Chain Probability

Event chain probability refers to the probability of various event chains caused by certain trigger factors (vehicle body fire, valve failure, tank rupture, brake failure, tire burst, rollover, scratch, impact, and rear-end collision), expressed as $H_{ij}$. For example, the probability of a fire–leakage event chain caused by a vehicle body fire is 0.0769, and the probability of an explosion event chain caused by a vehicle body fire is 0.0192. The probability values of each event chain can be obtained, as shown in Table S2 in the Supplementary material, according to the statistical results in Section 3.3.2. The case where the trigger factor is “others” and the event chain type is “others” is not studied here.

#### 4.1.3. Evacuation Trigger Probability

Evacuation trigger probability refers to the probability of triggering an evacuation event when a trigger factor results in an event chain, expressed as $E_{ij}$. For example, the probability of fire triggering an evacuation event caused by a vehicle body fire is 0.0135. The value of each evacuation trigger probability can be obtained, as shown in Table S3 in the Supplementary material, according to the statistical results of this study. The case where the trigger factor is “others” and the event chain type is “others” is not studied here.

### 4.2. Evacuation Event Consequence Calculation and Estimation

The rapid development of emergency relief becomes the main priority of government work after the occurrence of HCRTAs. To minimize the mortality rate, it is worthwhile to pay greater economic costs. However, the country’s public resources are limited, so it is necessary to make scientific and overall arrangements for the emergency rescue process. We should not only ensure the safety of people but also achieve successful rescue work [44] at a small economic cost. Theoretically, it is difficult to measure the value of human life with money. We will not advocate decision making based on the narrow benefit–cost method [45,46]. However, without a certain awareness of the costs in the process of an emergency evacuation, it is difficult to effectively promote the contribution of these costs to risk management [47]. The essence of cost calculation is the identification and measurement of accident consequences [48]. Therefore, this study uses the social cost of the evacuation process to reflect the consequences of evacuation events. Its calculation is shown in Equation (8):

(8) $$C=\frac{C_{1}+C_{2}}{G}\times 10^{8}$$

where C Indicates the social cost of evacuation events. $C_{1}$ refers to the labor loss value of personnel involved in emergency evacuation (yuan). $C_{2}$ is the cost of evacuation, transfer, and resettlement (yuan). G represents the gross national product in the year of measurement (yuan).

#### 4.2.1. Value of Labor Loss of Personnel Involved in Emergency Response

According to the emergency evacuation process in the National Emergency Response Plan [49], the main rescue personnel involved in the process of emergency evacuation and disposal of hazardous chemicals in road transportation accidents are firefighters, traffic police, government staff organizing evacuation, medical personnel, emergency experts, engineering rescue, military police, and environmental protection personnel. In this study, we only suggest the time cost of fire rescue personnel, public security traffic police, and government staff who organize evacuation (referred to as emergency evacuation personnel in this study) due to their participation in emergency rescue. The energy consumed by these personnel as a result of their involvement in the emergency evacuation and disposal of the accident is bound to curtail the chances of these personnel devoting themselves to other social activities. Therefore, the time cost of this personnel due to their participation in emergency evacuation is called the value of lost labor (considering that rescue personnel work an average of 8 h a day and 250 days a year). The calculation is shown in Equation (9):

(9) $$C_{1}=\frac{G_{p}\times M\times T_{f}}{8\times 250}$$

where $G_{p}$ refers to the per capita gross national product in the measurement year (yuan/person). M Indicates the number of people involved in accident rescue (person). $T_{f}$ indicates the time (h) that rescuers are involved in the emergency response.

#### 4.2.2. Personnel Evacuation and Resettlement Cost

The cost of personnel evacuation and resettlement primarily refers to the cost of the resettlement of uninjured or slightly injured personnel and the cost of evacuation and resettlement of affected surrounding residents during the evacuation process. The calculation is shown in Equation (10).

(10) $$C_{2}=\frac{C_{c}\times N\times T_{d}}{365\times 24}$$

where $C_{c}$ represents the per capita consumption level in the measurement year (yuan/person). N indicates the number of evacuees. $T_{d}$ refers to the time for the evacuated people to stay at the resettlement site (h).

### 4.3. Machine-Learning-Assisted Evacuation Event Classification

This study defined evacuation events according to the size of social risk. The value of evacuation probability of all possible scenarios ($F_{ij}$) is calculated according to Equation (7) in Section 4.1. According to the evacuation event statistics, the number of evacuees N, emergency evacuation personnel M, and emergency rescue time *T* are graded 1, 2, 3, or 4. Combining Equations (8)–(10) in Section 4.2, the social cost of each class is calculated (in the classification process of evacuation events, it is assumed that the time for rescuers to participate in emergency evacuation is equal to the time for evacuees to stay at the resettlement site, $T_{f}=T_{d}$). The results are shown in Table 5. The classification basis of evacuation number, emergency rescue number, and emergency rescue time are: the number of accidents including the number of evacuees, the number of accidents with the number of emergency rescue personnel, and the number of accidents with the emergency rescue time in the evacuation events are classified as level 1 according to the first 37%, level 2 according to the next 27%, level 3 according to the next 23%, and level 4 according to the remaining 13%. For example, in evacuation events, there are 30 types of accident information including the number of evacuees, 11 of them with N ≤ 100, accounting for 37%. There are 8 accidents with 100 < N ≤ 500, accounting for 27%. There are seven accidents with 500 < N ≤ 1000, accounting for 23%. There are four accidents with N > 1000, accounting for 13%.

**Figure 13:**
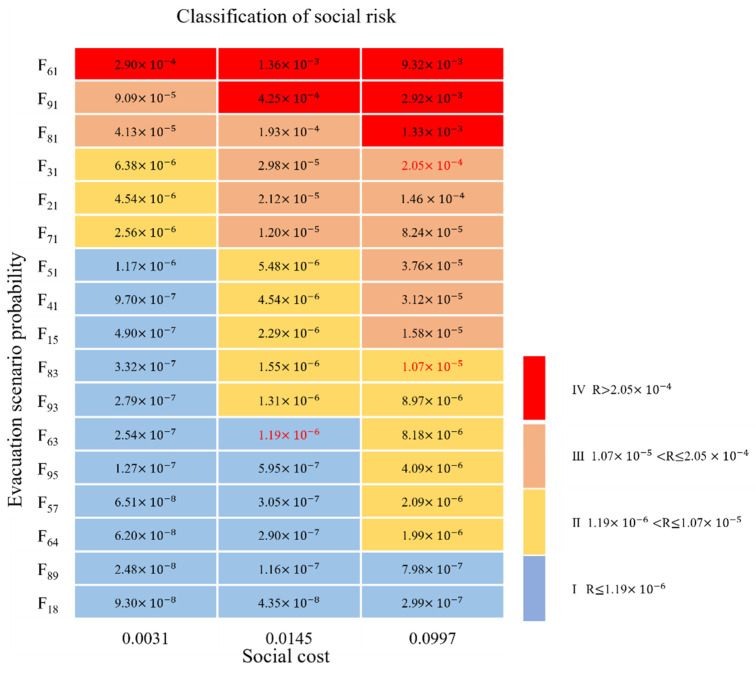
Social risk classification criteria for evacuation events.

According to the scenario evacuation probability ($F_{ij}$) and the social cost calculated in Table 5, the social risk is estimated in combination with Equation (6). The estimated social risk results are arranged from small to large. The first 37% is level I, the next 27% is level II, the next 23% is level III, and the last 13% is level IV. The classification results are shown in Figure 13.

**Figure 14:**
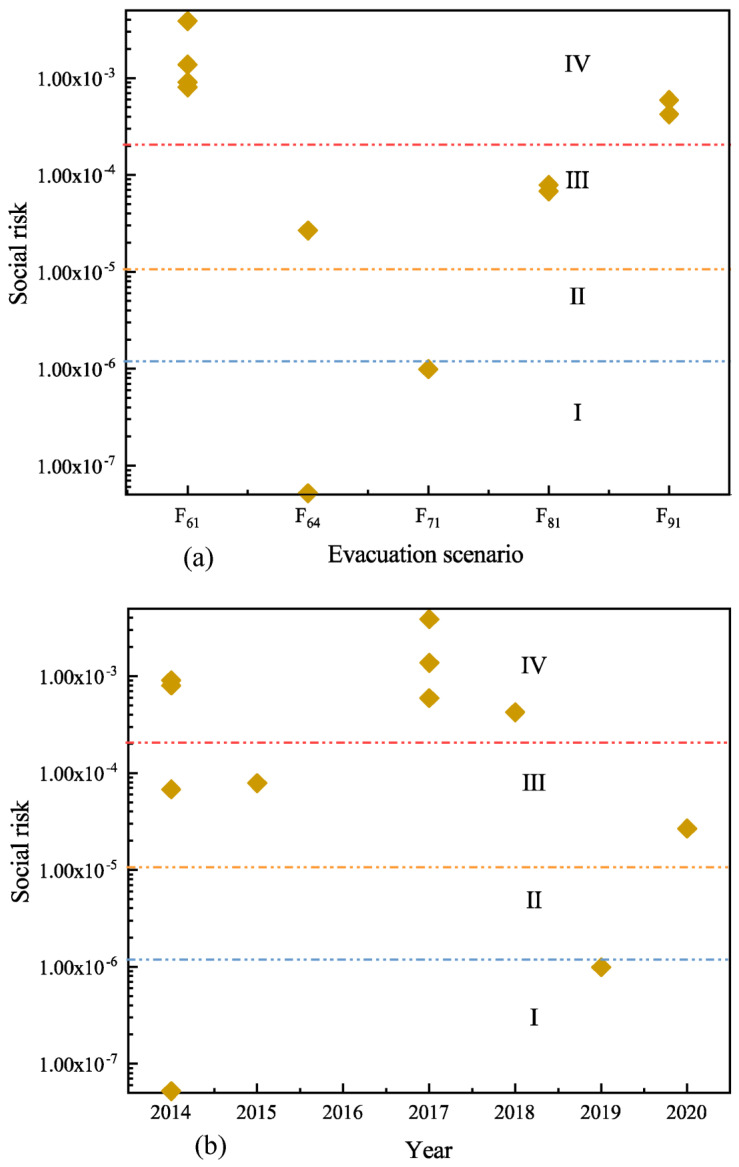
Social risk distribution of 11 evacuation events based on statistical data. (**a**) Social risk distribution of different evacuation scenarios. (**b**) Social risk distribution for different years.

In the 148 evacuation events, only 11 accidents have complete information on evacuation scenario probability, number of evacuees, number of emergency rescues, and emergency rescue time. As a result, these 11 accidents are analyzed, as shown in Figure 14. Evacuation scenario $F_{61}$ (the situation of personnel evacuation due to leakage caused by the rollover of hazardous chemical vehicles) shown in Figure 14a has a high social risk value, which is at level IV. The social risks arising from the evacuation scenario $F_{64}$ (the scenario of leakage–fire–explosion due to the overturning of hazardous chemical vehicles, which results in the evacuation of persons) are relatively dispersed and located in I and III. The social risks arising from evacuation scenarios $F_{81}$ (the leakage caused by the collision of hazardous chemical vehicles, which results in evacuation) and $F_{91}$ (the leakage caused by the rear-end of hazardous chemical vehicles, which results in evacuation) are relatively concentrated and located in III and IV, respectively. The social risk of evacuating persons due to a leakage caused by the impact of a hazardous chemical vehicle and the social risk of evacuating persons due to a leakage caused by the rear-end collision of a hazardous chemical vehicle are both relatively high. Figure 14b represents the changes in social risk for the above 11 accidents in several years. The social risk distribution of accidents in 2014–2019 is quite dispersed. The social risk of accidents in 2017 is relatively high, more concentrated in IV. If the social risk of the year is represented by the maximum value of the annual social risk, the social risk decreased year by year from 2017 to 2019 but increased in 2020. This trend is similar to the trend of the number of fatalities, accident rates, and mortality in Figure 1.

Fewer incidents containing complete evacuation information can be collected. Therefore, in this study, the abovementioned 11 incidents containing complete evacuation information are used as the primary data, and machine learning is used to supplement the missing information that cannot be obtained. The number of evacuees represents the ease of handling accidents; emergency rescue personnel and emergency rescue time represent the ability to handle the accident. Based on this, a complete connected neural network model is developed to fit the data to obtain an approximate solution. It includes input, output, and hidden layers. It has strong approximation ability, classification ability, and learning convergence speed. When the input pattern vector is extended to the hidden layer space, the function set constructs an arbitrary basis, so that the original nonseparable problem in the low dimensional space is transformed into an approximate linear separable problem in the high dimensional space, and the approximation of any function with arbitrary precision is realized [50,51,52].

**Figure 15:**
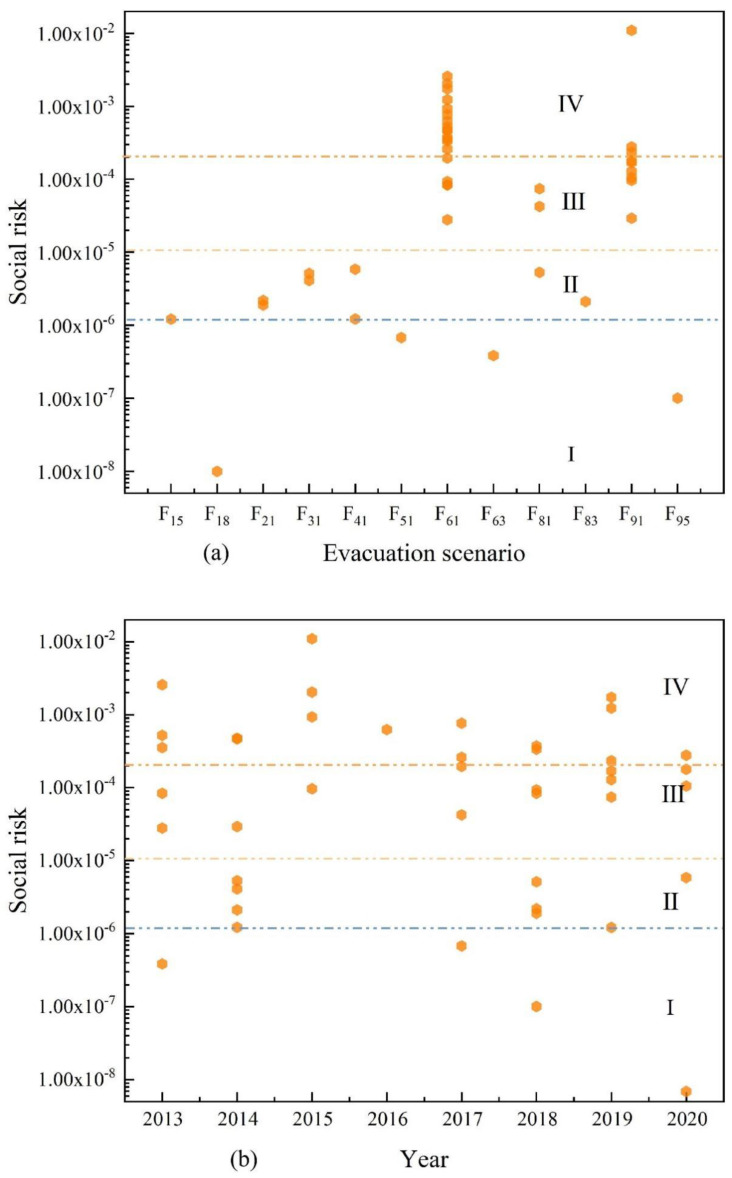
Machine learning social risk distribution of 43 evacuation events. (**a**) Social risk distribution for different evacuation scenarios. (**b**) Social risk distribution for different years.

In this study, emergency rescue personnel and emergency rescue time are utilized as inputs, and the predicted number of evacuees is utilized as output. Five layers of hidden space are constructed. Among 148 evacuation events, only 43 accidents contain the complete information of evacuation scenario probability, emergency rescue number, and emergency rescue time simultaneously (excluding 11 accidents with complete information of evacuation scenario probability, evacuation number, emergency rescue number, and emergency rescue simultaneously). The 43 accidents are analyzed, as demonstrated in Figure 15. The social risks developing from evacuation scenarios $F_{61}$ and $F_{91}$ have a higher probability compared with other scenarios in Figure 15a. This conclusion is the same as Figure 14a. In Figure 15b, the distribution of social risk values for each year is more dispersed and distributed at all levels, but the complete social risk of 2015 is relatively high. Therefore, the scenario of hazardous chemical vehicles causing leakage from collision leading to evacuation and the scenario of hazardous chemical vehicles causing leakage from rear-end leading to evacuation deserve the attention of relevant regulatory authorities, enterprises, and researchers.

## 5. Framework for Improving the Emergency Capacity of Communities along with the Road Transportation of Hazardous Chemicals

The recent regulations and standards for road transportation of hazardous chemicals in China primarily include the Rules for Road Transportation of Hazardous Goods [53] and the Measures for Road Transportation Safety Management of Hazardous Goods [54]. The rules for road transportation, vehicles and equipment, driver training, and parking of hazardous chemicals are regulated. In addition, the limitations on the route of vehicles transporting hazardous goods are partially discussed. It is observed that the safety management of road transportation of hazardous chemicals in China focuses on the potential to effectively prevent the leakage of hazardous chemicals from vehicles, but clear standards and impact on reducing the risk to public living near the traffic route after the leakage of hazardous chemicals are lacking [55]. However, while top–down standards are needed, bottom–up policies at the local level contribute to the impetus for the implementation of disaster reduction strategies and disaster management [56]. Commonly, if the community can perform emergency-related work effectively, it is more likely to take effective measures to reduce accident risks [57].

**Figure 16:**
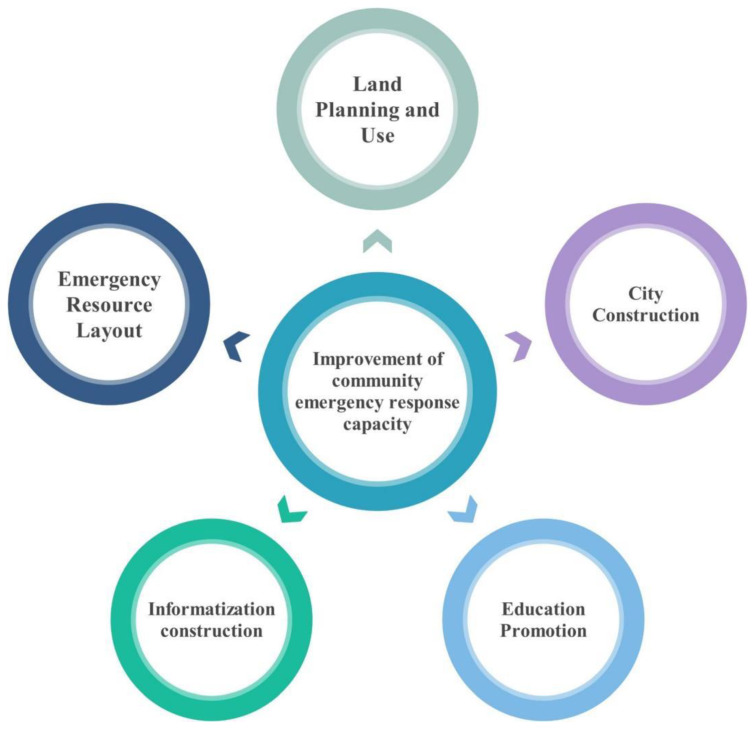
Framework for improving the emergency capacity of communities along with the road transportation of hazardous chemicals.

Based on the analysis of the characteristics of China’s road transportation management of hazardous chemicals from 2012 to 2020, combined with advanced experience in enhancing the emergency management capacity of communities at home and abroad [57,58,59,60], this study proposes a structure for enhancing the emergency capacity of communities as well as the road transportation of hazardous chemicals in China, as demonstrated in Figure 16. The emergency capacity of the community should be enhanced, and districts should be improved in terms of land use, city construction, education promotion, information construction, and emergency resource layout to reduce the risks faced by the public as well as the road transportation of dangerous chemicals.

### 5.1. Land Planning and Use

To reduce social risk, essential standards and regulations regarding the management of hazardous chemicals need to be updated in a timely and dynamic manner. The Chinese national standard method for determining the external safety distance of hazardous chemical production and storage facilities [61] specifies the method for estimating the external safety distance of hazardous chemical production and storage facilities using various circumstances. Therefore, when planning and developing the expressway, the local government should approve scientific and logical methods to estimate the spread range of hazardous chemical vehicles, to determine the layout and safety distance between the expressway and surrounding communities and factories. The safety distance should be increased in densely populated and industrial regions where accidents may cause secondary injury. Safety standards for essential regions should be enhanced, and necessary funds should be invested in them. Residents of nearby communities can be invited to participate in the land planning of the community section, which is conducive to enhancing the rationality of the planning [62].

### 5.2. City Construction

To maximize economic and safety benefits, the site selection of hazardous chemical infrastructures, such as chemical parks, hazardous chemical transportation, and storage centers, is examined and reasonably planned. The types, supply and demand, and risk characteristics of hazardous chemicals in the region should be clarified, and the location of the infrastructure be selected with the aid of GIS spatial analysis technology in combination with the industrial pattern, environmental pollution, population, transportation, land-use rate, urban planning, emergency evacuation capacity, and other factors [63]. Local public security authorities can develop special transport lanes at specific times so hazardous chemical transport vehicles in specific transport periods and transport roads are effectively monitored. The time and lane transportation should be selected to prevent the peak period of vehicle flow. The region where the road surface is smooth and in good condition and away from the dense flow of drinking water sources should be selected. Simultaneously, the accident-prone, high-incidence road should focus on supervision and develop warning signs. Road infrastructure and the surrounding environment should be enhanced [64].

### 5.3. Education Promotion

Based on the characteristics of hazardous chemical road transportation, local government departments can popularize the awareness of HCRTAs and the basic skills for accident prevention and community evacuation via various forms such as online public classes, new media live broadcasts, and online interviews [65]. Local government departments should also cooperate with permanent institutions qualified for emergency training investigation onsite or online public experience activities for HCRTAs. Government departments can develop a “Forum on Community Evacuation due to road transportation accidents due to hazardous chemicals” to obtain successful cases of community evacuation due to hazardous chemical accidents at home and abroad, analyze and summarize their successful experiences, and primary practices for public learning in the community [59]. Publicity and educational products such as books, teaching materials, animation, games, movies, and dramas should be developed for various communities to deal with HCRTAs. Awareness of hazardous chemicals via popular media (such as WeChat, Tik Tok, and Weibo) enhancing the actual effect of publicity and education should be disseminated [63]. Considering the neighborhood’s older population and educational level, community radio can be utilized to broadcast first aid awareness and emergency skills for HCRTAs. The methods in which the community receives public education on awareness of HCRTAs should be expanded continually.

### 5.4. Informatization Construction

The relevant administrative departments can develop a national information platform for the management of hazardous chemical transport to promote cross-regional and cross-departmental information exchange. They can also make full use of Beidou positioning technology, 5G platform, big data, Internet of Things, artificial intelligence algorithms, and other advanced technologies to correlate information related to the safe environment of sensitive areas along the route with the safety characteristics and quantity of hazardous chemicals transported and complete the dynamic assessment of risks along the hazardous chemical transportation route [55]. Appropriate routes for drivers should be recommended according to the level of risk. Professional hazardous chemical emergency evacuation management systems and portals applicable to hazardous chemical practitioners and public applications in surrounding communities should be developed. For example, once a driver encounters a hazardous chemical emergency, the driver can quickly locate and alert the police. It buys rescue time for the rescuers and can help them deal with the accident and carry out rescue efforts quickly [63]. We recommend building a national “one network” of dangerous chemical transport enterprise registration information, electronic waybill supervision information, road transport management information, tank inspection information, road risk information, and dangerous chemical emergency rescue information data [66].

### 5.5. Emergency Resource Layout

Due to the special nature of rescues for HCRTAs, it is considered that the Ministry of Emergency Management should improve, and essential departments should participate in developing an organization and coordination body for emergency evacuation, which should become a branch of the national and local emergency evacuation system [67]. In accident-prone communities along roads where transportation of hazardous chemicals is routed, considering the characteristics of HCRTAs and in combination with community conditions, community emergency rescue resource pools, medical treatment points, and emergency fire stations should be developed. There are several types of hazardous chemicals, and their protective emergency measures vary. Therefore, it is appropriate that each region should develop targeted chemical protection equipment standards according to the characteristics of local transportation of hazardous chemicals and uniformly equip by the community. Simultaneously, a family emergency manual, emergency equipment, and a list of recommendations to promote the usage of family emergency kits are being developed and published. Based on the internal emergency rescue forces of large- and medium-sized enterprises, community fire teams can also be developed together with government community staff and community public representatives, and onsite or desktop drills can be conducted regularly. Complete usage of new technologies and methods such as the Internet, big data, and we media, as well as traditional methods such as emergency broadcasting, loudspeaker, and whistleblowing, are recommended to actively promote the development experience of “village to village” emergency broadcasting. Cooperation of the community with neighboring supermarkets and enterprises to conduct emergency material agreement reserves should be promoted [66].

## 6. Discussion

It is essential to note certain challenges in conducting accident information statistics and a significant proportion of accidents lack evacuation information. To make the evacuation classification model reliable, complete incident information is required. HCRTAs in the description of relevant accident information should be obvious after alert range, number of evacuees (evacuation range, nearby population density), evacuation time, number of people involved in the emergency rescue, emergency disposal time, evacuation personnel resettlement time, and other information. This study utilizes the collected data as the benchmark for statistical analysis, so there may be bias in the evaluated results. The following is an analysis of the potential causes of bias:

(1) During the statistical process, the evacuation events triggered by a leakage event chain were compared with the evacuation events triggered by other event chains. The primary reason is that after a leakage event chain type of accident, more evacuations are required. In addition, a leakage–fire–explosion event chain type of accident commonly includes casualties, and the public is focused on the casualties rather than the number of evacuees, resulting in incomplete evacuation information and influencing the probability value of evacuation scenarios.

(2) An accident that caused injury to the perimeter of the accident site requires more emergency personnel to evacuate a similar number of persons compared with an accident that causes injury within its perimeter. This is because if evacuation occurs during the stage before an accident causes injury, people can evacuate in an orderly manner on their own using emergency information dissemination, decreasing the need for emergency rescue personnel. Many people will be evacuated, and just a small number of people need emergency rescue. If people within the perimeter of the accident are unable to evacuate by themselves due to various reasons when the accident has caused injury, the number of emergency rescue personnel will increase, and the emergency rescue time may increase. When exploring the relationship between the number of evacuees and the required emergency rescue personnel, emergency rescue time should be noted.

(3) When constructing an evacuation event classification model based on social risk assessment, the consequences of an evacuation event should have considered the social costs of remediation of environmentally sensitive factors such as river system, soil, and vegetation caused by hazardous chemical spills. Compared with hazardous chemical accidents in industrial parks, the environmental pollution problems caused by hazardous chemical accidents during transportation need to be studied more. This is due to factors such as the uncertainty of the geography of the transportation process and the diversity of land use types. However, due to the deficient data sources, the description of the extent of damage to the surrounding environment was less, and the information collected was insufficient to support the model construction, and attention was paid to collecting information about this aspect in future studies.

## 7. Conclusions

This study reveals the recent characteristics of HCRTAs and emergency evacuation responses in China. Since 2014 and 2016, the fatality rate and accident frequency of HCRTAs in China have gradually decreased. The frequency of accidents in winter is even higher than that in summer, but the death rate is lower than that in summer. There are varieties in the frequency of HCRTAs between the eastern and western regions of China. The trigger factors of HCRTAs are primarily rollover and rear-end collision.

The results of the risk classification of evacuation events show that the scenario of evacuation caused by the overturning of a hazardous chemical vehicle has the highest social risk ratio. This is followed by the scenario in which the spill is caused by the impact of a hazardous chemical vehicle, and the scenario in which the spill is caused by the rear-end of a hazardous chemical vehicle, which leads to the evacuation of people. If the maximum social risk in each year demonstrates the social risk of this year, the social risk will decrease year by year from 2017 to 2019 but will increase in 2020. This trend is like that of the deaths, accident frequency, and mortality rate in various years.

At present, several countries attach high importance to the safety management of road transportation of hazardous chemicals. However, research on public emergency protection actions and guidance strategies for the communities along the transportation route after a road transportation accident involving hazardous chemicals is insufficient to meet the requirement of public safety. This study can be used to construct evacuation scenarios for communities along different hazardous chemical road transport routes to help make decisions when faced with future hazardous chemical transport accidents.

## Figures and Tables

**Table 1:** Evolution process and severity of different event chains.

Category of Accidents	Frequency of Accidents	Percentage of Total Accidents	Mortality Rate
Leakage	2176	81.90%	6%
Leakage–Explosion	2	0.08%	0%
Leakage–Fire	124	4.67%	27%
Leakage–Fire–Explosion	44	1.66%	427%
Fire	148	5.57%	18%
Fire–Explosion	31	1.17%	68%
Fire–Leakage	17	0.64%	0%
Explosion	28	1.05%	75%
Explosion–Fire	14	0.53%	107%
Others	73	2.75%	7%

**Table 2:** Grade distribution of accident deaths.

Death Toll Grade (People)	Frequency of Accidents	Percentage of Total Accidents
No fatalities	2488	93.60%
1–2	149	5.61%
3 or more	21	0.79%

**Table 3:** Grade distribution of accident injuries.

Injury Grade (People)	Frequency of Accidents	Percentage of Total Accidents
Uninjured	2244	84.42%
1–2	351	13.21%
3–9	50	1.88%
10 or more	13	0.49%

**Table 4:** Typical of HCRTAs.

Year	Description of Accidents	Time	Category of Accidents	Trigger Factor	Road Types	Road Name	Death Tolls	Hazardous Chemicals	Main Causes of Accidents
2020	“6·13” explosion accident of Wenling tank car	16:46 on 13 June	Leakage–Fire–Explosion	Rollover	Expressway	Shen Hai Expressway	20	Liquefied Petroleum Gas	Overspeed rollover
2017	Explosion accident of the Hebei Zhang shi Expressway Tunnel	6:30 on 23 May	Fire–Explosion	Car body fire	Expressway	Zhang Shi Expressway	15	Sodium chlorate	Spontaneous combustion of rubber tires, causing sodium chlorate explosion
2014	“7·19” particularly serious accident in the Shaoyang section of the Shanghai Kunming Expressway in Hunan	6:30 on 23 May	Leakage–Fire–Explosion	Rear-end collision	Expressway	Shanghai Kunming Expressway	54	Ethanol	The rear-end collision between the bus and truck caused leakage, combustion, and explosion
2014	“3.1” accident in the Yanhou tunnel in the Shanxi Jincheng section of the Jinji Expressway	14:45 on 1 March	Leakage–Fire–Explosion	Rear-end collision	Expressway	Jin Ji Expressway	40	Methanol	Two methanol trucks hit the rear-end, and methanol leakage ignited other dangerous chemical vehicles and coal carriers
2012	Shaanxi Yan’an “8·26” particularly serious road traffic accident on the Baomao Expressway	2:31 on 26 August	Leakage–Fire–Explosion	Rear-end collision	Expressway	Bao Mao Expressway	36	Methanol	The bus did not brake in time to avoid impact on rear-end of the methanol tanker; methanol leakage caused fire and explosion
2012	Guangzhou oil tanker explosion and combustion accident	4:30 on 29 June	Leakage–Fire–Explosion	Rear-end collision	Expressway	Riverside Expressway	20	Solvent oil	After the rear-end collision between the truck and the tanker truck, it ignited the goods stacking plant and shed under the viaduct

**Table 5:** Evacuation information classification standard.

Grade	Number of Evacuees	Emergency Responders	Emergency Rescue Time	Social Costs
1	N ≤ 100	M ≤ 15	T ≤ 4	C ≤ 0.0031
2	100 < N ≤ 500	15 < M ≤ 25	4 < T ≤ 7	0.0031 < C ≤ 0.0145
3	500 < N ≤ 1000	25 < M ≤ 50	7 < T ≤ 24	0.0145 < C ≤ 0.0997
4	N > 1000	M > 50	T > 24	C > 0.0997

## Data Availability

Not applicable.

## Supplementary Materials

The following supporting information can be downloaded at: https://www.mdpi.com/article/10.3390/ijerph192215182/s1. Table S1. The proportion of accident frequency of different trigger factors. Table S2. Probability of different event chains caused by different trigger factors in road transportation accidents of hazardous chemicals. Table S3. Different trigger factors in evacuation events lead to different event chain probabilities.

## References

[1] Liu Y.H. Operation report of China’s hazardous chemicals logistics industry in 2021. Proceedings of the China Chemical Logistics Industry Annual Meeting.

[2] Huang X., Wang X., Pei J., Xu M., Huang X., Luo Y. (2018). Risk assessment of the areas along the highway due to hazardous material transportation accidents. Nat. Hazards.

[3] China Emergency Broadcasting (2019). Major Liquid Nitrogen Leakage Accident in Huai’an, Jiangsu. http://www.cneb.gov.cn/2019/11/11/VIDE1573430281407624.shtml.

[4] Netease (2021). 2020 Summary of Major Dangerous Chemical Transportation Accidents in China and Abroad. https://www.163.com/dy/article/G0L7GM6K0538FBWT.html.

[5] Ren C.G., Yuan X.J., Wang J., Zhang X., Li J. (2012). 2012 International symposium on safety science and technology study on emergency response rank mode of flammable and explosive. Procedia Eng..

[6] Cao J., Shi S.L., Lu Y., Wang Y., Peng J.H. (2020). Analysis on tank transportation accidents of hazardous chemicals from 2013 to 2018. China Saf. Sci. J..

[7] Liu Y., Fan L.-S., Li X., Shi S.-L., Lu Y. (2020). Trends of hazardous material accidents (HMAs) during highway transportation from 2013 to 2018 in China. J. Loss Prev. Process Ind..

[8] Yang J., Li F., Zhou J., Zhang L., Huang L., Bi J. (2010). A survey on hazardous materials accidents during road transport in China from 2000 to 2008. J. Hazard. Mater..

[9] Li Y., Ping H., Ma Z.-H., Pan L.-G. (2014). Statistical analysis of sudden chemical leakage accidents reported in China between 2006 and 2011. Environ. Sci. Pollut. Res..

[10] Chen W.Y., Wang Y., Li Q.H. (2020). Study on risk prediction model of road transportation of hazardous chemicals. J. Saf. Environ..

[11] Shen X., Wei S. (2021). Severity analysis of road transport accidents of hazardous materials with machine learning. Traffic Inj. Prev..

[12] Liu Z., Li X., Chen X. (2019). Evacuation traffic management under diffusion of toxic gas based on an improved road risk level assessment Method. Complexity.

[13] Cao P.B. (2018). Study on Scenarios Construction and Emergency Management of Road Transportation Accident of Hazardous Chemicals. Master’s Dissertation.

[14] Oggero A., Darbra R.M., Munoz M., Planas E., Casal J. (2006). A survey of accidents occurring during the transport of hazardous substances by road and rail. J. Hazard. Mater..

[15] Wu Z.Z., Duo Y.Q., Liu M., Gao J.D., Wei L.J. (2004). Study on risk analysis and evaluation method of road transportation of dangerous goods. J. Basic Sci. Eng..

[16] Verter V., Kara B.Y. (2001). A GIS-based framework for hazardous materials transport risk assessment. Risk Anal..

[17] Fabiano B., Curro F., Reverberi A., Pastorino R. (2005). Dangerous good transportation by road: From risk analysis to emergency planning. J. Loss Prev. Process Ind..

[18] Guo X.L., Li J. (2006). Research on risk measurement model of hazardous goods transportation path based on accident classification. China Saf. Sci. J..

[19] AlRukaibi F., Alrukaibi D., Alkheder S., Alojaiman S., Sayed T. (2018). Optimal route risk-based algorithm for hazardous material transport in Kuwait. J. Loss Prev. Process Ind..

[20] Xie Y., Lu W., Wang W., Quadrifoglio L. (2012). A multimodal location and routing model for hazardous materials transportation. J. Hazard. Mater..

[21] Reniers G.L.L., Jongh K.D., Gorrens B., Lauwers D., Leest M.V., Witlox. F. (2010). Transportation risk analysis tool for hazardous substances (TRANS)-A user-friendly, semi-quantitative multi-mode hazmat transport route safety risk estimation methodology for Flanders. Transport. Res. Part D-Transport. Environ..

[22] Bubbico R., Di Cave S., Mazzarotta B. (2004). Risk analysis for road and rail transport of hazardous materials: A simplified approach. J. Loss Prev. Process Ind..

[23] Zografos K.G., Vasilakis G.M., Giannouli I.M. (2000). Methodological framework for developing decision support systems (DSS) for hazardous materials emergency response operations. J. Hazard. Mater..

[24] Frank W.C., Thill J.-C., Batta R. (2000). Spatial decision support system for hazardous material truck routing. Transp. Res. Part C Emerg. Technol..

[25] Torretta V., Raboni M., Copelli S., Urbini G. (2013). Application of a decision support system to the transport of hazardous materials. Environ. Eng. Manag. J..

[26] Bubbico R., Maschio G., Mazzarotta B., Milazzo M.F., Parisi E. (2006). Risk management of road and rail transport of hazardous materials in Sicily. J. Loss Prev. Process Ind..

[27] Milazzo M.F., Lisi R., Giuseppe M., Maschio G., Antonion G., Spadoni G. (2010). A study of land transport of dangerous substances in Eastern Sicily. J. Loss Prev. Process Ind..

[28] Van Raemdonck K., Macharis C., Mairesse O. (2013). Risk analysis system for the transport of hazardous materials. J. Saf. Res..

[29] Cui Y.H. (2013). Research on Information Processing Technology of Transportation Safety Monitoring of Hazardous Chemicals Tank Trucks. Ph.D. Dissertation.

[30] Torretta V., Rada E.C., Schiavon M., Viotti P. (2017). Decision support systems for assessing risks involved in transporting hazardous materials: A review. Saf. Sci..

[31] Dumrongpokaphan T., Kreinovich V. (2017). Kuznets Curve: A Simple Dynamical System-Based Explanation.

[32] Chai M.I. (2019). Identification and Warning of Fatigue Status of Coach Drivers. Ph.D. Dissertation.

[33] Hou J., Gai W.-M., Cheng W.-Y., Deng Y.-F. (2020). Hazardous chemical leakage accidents and emergency evacuation response from 2009 to 2018 in China: A review. Saf. Sci..

[34] (2021). Classification and Code of Dangerous Goods.

[35] The Accident Investigation Team (2020). Investigation Report on Major Explosion Accident of “6.13” Liquefied Petroleum Gas Transportation Tank Truck in Wenling Section of Shen Hai Expressway. https://yjt.zj.gov.cn/art/2020/12/31/art_1228978417_59031875.html.

[36] Hou S.Y., Wang Z., Luan X.Y., Liu Y.C., Gong Y.W., Guo C.C., Wang S.R., Zhang B. (2021). Analysis of tank truck explosion accident in Wenling, Zhejiang Province. Natural Science Edition. J. Nanjing Tech Univ..

[37] Xu X.H. (2020). Doubts and Conjectures about the Explosion of a Tank Car in Wenling, Zhejiang Province. Research on Safety Science and Emergency Management. https://mp.weixin.qq.com/s/XfvEacv-6-5ewkBwbf7mHw.

[38] Order of the State Council of the People’s Republic of China (2011). Regulations on Highway Safety Protection. http://www.gov.cn/zwgk/2011-03/14/content_1824439.htm.

[39] Waters M.C. (2016). Life after hurricane katrina: The resilience in survivors of katrina (RISK) Project. Sociol. Forum.

[40] Saat M.R., Werth C.J., Schaeffer D., Yoon H., Barkan C.P. (2014). Environmental risk analysis of hazardous material rail transportation. J. Hazard. Mater..

[41] ISDR (2002). Living with Risk: A Global Review of Disaster Reduction Initiatives.

[42] Francyelly G.C., Barbara S.B., Anna S.P.P., Rui A.R.R. (2016). Methodological aspects for modeling the environmental risk of transporting hazardous materials by road. Transp. Res. Part D.

[43] Erhan E., Vedat V. (1998). Modeling of transport risk for hazardous materials. Oper. Res..

[44] Liu H.C. (2015). Disaster emergency rescue strategy based on disaster relief cost analysis. J. Zhengzhou Inst. Aeronaut. Ind..

[45] Tapsell S.M., Penning-Rowsell E.C., Tunstall S.M., Wilson T.L. (2002). Vulnerability to flooding: Health and social dimensions. Philos. Trans. R. Soc. Lond. Ser. A Math. Phys. Eng. Sci..

[46] Handmer J. (2002). The chimera of precision: Inherent uncertainties in disaster loss assessment. Int. J. Disaster Risk Reduct..

[47] Edmund P.R., Theresa W. (2006). Gauging the impact of natural hazards: The pattern and cost of emergency response during flood events. Trans. Inst. Br. Geogr..

[48] Markku V.P.A., Erkki U.R., Jorma S., Mari A.P., Tuula R., Kari V. (1996). The accident consequence tree method and its application by real-time data collection in the Finnish furniture industry. Saf. Sci..

[49] The State Council (2006). Master State Plan for Rapid Response to Public Emergencies. http://www.gov.cn/yjgl/2006-01/08/content_21048.htm.

[50] Chen J., Li Q., Wang H., Deng M. (2019). A Machine Learning Ensemble Approach Based on Random Forest and Radial Basis Function Neural Network for Risk Evaluation of Regional Flood Disaster: A Case Study of the Yangtze River Delta, China. Int. J. Environ. Res. Public Health.

[51] Li W., Bazant M.Z., Zhu J. (2021). A physics-guided neural network framework for elastic plates: Comparison of governing equations-based and energy-based approaches. Comput. Methods Appl. Mech. Eng..

[52] Lykkegaard M.B., Dodwell T.J., Moxey D. (2021). Accelerating uncertainty quantification of groundwater flow modelling using a deep neural network proxy. Comput. Methods Appl. Mech. Eng..

[53] (2018). Rules for Road Transport of Dangerous Goods.

[54] Ministry of Transport of the People’s Republic of China (2019). Measures for the Administration of Road Transport Safety of Dangerous Goods. https://gkml.samr.gov.cn/nsjg/bgt/202106/t20210625_331531.html.

[55] Liu R.C., Liu A.Q., Han H.C. (2020). Risk management experience of international road transportation dangerous chemicals. Labor Prot..

[56] Laurie P. (2003). Disaster Management and Community Planning, and Public Participation: How to Achieve Sustainable Hazard Mitigation. Nat. Hazards.

[57] Kambod A.H., Yasamin O.I. (2020). From “Earthquake and safety” school drills to “safe school-resilient communities”: A continuous attempt for promoting community-based disaster risk management in Iran. Int. J. Disaster Risk Reduct..

[58] Barbara R., Johnston K.A., Maureen T., Ryan M. (2020). Community engagement for disaster preparedness: A systematic literature review. Int. J. Disaster Risk Reduct..

[59] Sheng D.P. (2018). Study on the Improvement of Emergency Management Ability of Sanyuan Community in Chengdu. Master’s Dissertation.

[60] Qiao Z. (2017). Exploration on the path of urban and rural community governance capacity construction. China Emerg. Rescue.

[61] (2019). Method for Determining External Safety Distance of Hazardous Chemicals Production Units and Storage Installations.

[62] Kristen M.T. (2014). The district of North Vancouver’s landslide management strategy: Role of public involvement for determining tolerable risk and increasing community resilience. Nat. Hazards.

[63] Xiao J.H., Yang X.C. (2020). Analysis and Research on road transportation accidents of hazardous chemicals in China. J. Political Sci. Law.

[64] Lv H., Zhu W., Peng M. (2017). Analysis and Countermeasures of transportation accidents of dangerous chemicals in Beijing. Mod. Occup. Saf..

[65] China National Disaster Reduction Commission (2020). Notice on Strengthening the Construction of Grass-Roots Emergency Response Capacity and Doing a Good Job in the Relevant Work of 2020 National Disaster Prevention and Reduction Day. https://www.mem.gov.cn/gk/tzgg/tz/202004/t20200414_349962.shtml.

[66] Safety Production Committee of the State Council (2020). National Centralized Regulation Scheme for Road Transportation Safety of Hazardous Chemicals. https://www.mem.gov.cn/xw/ztzl/2020/qmjqwhpaqscgz/bsap/202009/t20200910_361688.shtml.

[67] Liu J. (2018). Enlightenment of foreign dangerous goods road transportation safety management. Labor Prot..

