# Supplementary material for: Hazardous Chemicals Road Transportation Accidents and the Corresponding Evacuation Events from 2012 to 2020 in China: A Review
Source: Int J Environ Res Public Health. 2022 Nov 17;19(22):15182. doi: 10.3390/ijerph192215182 (PMC9690497; doi:10.3390/ijerph192215182)
Supplement: Supplementary file 1 [file ijerph-19-15182-s001.zip › ijerph-1994353-supplementary.pdf]

The values of an accident trigger probability, event chain probability, and evacuation trigger probability are shown in Table S1, Table S2, and Table S3.

Table S1. The proportion of accident frequency of different trigger factors.

Variable I_i	Description	Probability value
I_1	Probability of car body fire	0.0196
I_2	Probability of valve failure	0.0448
I_3	Probability of tank rupture	0.0399
I_4	Probability of brake failure	0.0173
I_5	Probability of tire burst	0.0429
I_6	Probability of rollover	0.3002
I_7	Probability of scraped	0.0267
I_8	Probability of impact	0.1535
I_9	Probability of rear-end collision	0.2528

Table S2. Probability of different event chains caused by different trigger factors in road transportation accidents of hazardous chemicals.

Variable H_{ij}	Description	Probability value
H_{11}	Probability of leakage caused by vehicle body fire	0.0000
H_{12}	Probability of leakage-explosion caused by vehicle body fire	0.0000
H_{13}	Probability of leakage-fire caused by vehicle body fire	0.0577
H_{14}	Probability of leakage-fire-explosion caused by vehicle body fire	0.0000
H_{15}	Probability of fire caused by vehicle body fire	0.5962
H_{16}	Probability of fire-explosion caused by vehicle body fire	0.0962
H_{17}	Probability of fire-leakage caused by vehicle body fire	0.0769
H_{18}	Probability of explosion caused by vehicle body fire	0.0192
H_{19}	Probability of explosion-fire caused by vehicle body fire	0.0192
H_{21}	Probability of leakage caused by valve failure	0.9664
H_{22}	Probability of leakage-explosion caused by valve failure	0.0000
H_{23}	Probability of leakage-fire caused by valve failure	0.0252
H_{24}	Probability of leakage-fire-explosion caused by valve failure	0.0000
H_{25}	Probability of fire caused by valve failure	0.0000
H_{26}	Probability of fire-explosion caused by valve failure	0.0000
H_{27}	Probability of fire-leakage caused by valve failure	0.0000
H_{28}	Probability of explosion caused by valve failure	0.0084
H_{29}	Probability of explosion-fire caused by valve failure	0.0000
H_{31}	Probability of leakage caused by tank rupture	0.9528
H_{32}	Probability of leakage-explosion caused by tank rupture	0.0094
H_{33}	Probability of leakage-fire caused by tank rupture	0.0377
H_{34}	Probability of leakage-fire-explosion caused by tank rupture	0.0000
H_{35}	Probability of fire caused by valve failure	0.0000
H_{36}	Probability of fire-explosion caused by tank rupture	0.0000
H_{37}	Probability of fire-leakage caused by tank rupture	0.0000
H_{38}	Probability of explosion caused by tank rupture	0.0000
H_{39}	Probability of explosion-fire caused by tank rupture	0.0000
H_{41}	Probability of leakage caused by brake failure	0.8913
H_{42}	Probability of leakage-explosion caused by brake failure	0.0000
H_{43}	Probability of leakage-fire caused by brake failure	0.0000
H_{44}	Probability of leakage-fire-explosion caused by brake failure	0.0217
H_{45}	Probability of fire caused by brake failure	0.0435
H_{46}	Probability of fire-explosion caused by brake failure	0.0435
H_{47}	Probability of fire-leakage caused by brake failure	0.0000
H_{48}	Probability of explosion caused by brake failure	0.0000
H_{49}	Probability of explosion-fire caused by brake failure	0.0000

H_{51}	Probability of leakage caused by tire burst	0.4336
H_{52}	Probability of leakage-explosion caused by tire burst	0.0000
H_{53}	Probability of leakage-fire caused by tire burst	0.0354
H_{54}	Probability of leakage-fire-explosion caused by tire burst	0.0354
H_{55}	Probability of fire caused by tire burst	0.3097
H_{56}	Probability of fire-explosion caused by tire burst	0.0354
H_{57}	Probability of fire-leakage caused by tire burst	0.0708
H_{58}	Probability of explosion caused by tire burst	0.0000
H_{59}	Probability of explosion-fire caused by tire burst	0.0000
H_{61}	Probability of leakage caused by rollover	0.9035
H_{62}	Probability of leakage-explosion caused by rollover	0.0025
H_{63}	Probability of leakage-fire caused by rollover	0.0401
H_{64}	Probability of leakage-fire-explosion caused by rollover	0.0100
H_{65}	Probability of fire caused by rollover	0.0125
H_{66}	Probability of fire-explosion caused by rollover	0.0013
H_{67}	Probability of fire-leakage caused by rollover	0.0000
H_{68}	Probability of explosion caused by rollover	0.0013
H_{69}	Probability of explosion-fire caused by rollover	0.0013
H_{71}	Probability of leakage caused by scraped	0.9155
H_{72}	Probability of leakage-explosion caused by scraped	0.0000
H_{73}	Probability of leakage-fire caused by scraped	0.0282
H_{74}	Probability of leakage-fire-explosion caused by scraped	0.0141
H_{75}	Probability of fire caused by scraped	0.0141
H_{76}	Probability of fire-explosion caused by scraped	0.0000
H_{77}	Probability of fire-leakage caused by scraped	0.0000
H_{78}	Probability of explosion caused by scraped	0.0000
H_{79}	Probability of explosion-fire caused by scraped	0.0000
H_{81}	Probability of leakage caused by impact	0.8554
H_{82}	Probability of leakage-explosion caused by impact	0.0000
H_{83}	Probability of leakage-fire caused by impact	0.0515
H_{84}	Probability of leakage-fire-explosion caused by impact	0.0147
H_{85}	Probability of fire caused by impact	0.0123
H_{86}	Probability of fire-explosion caused by impact	0.0147
H_{87}	Probability of fire-leakage caused by impact	0.0000
H_{88}	Probability of explosion caused by impact	0.0000
H_{89}	Probability of explosion-fire caused by impact	0.0074
H_{91}	Probability of leakage caused by rear-end collision	0.8586
H_{92}	Probability of leakage-explosion caused by rear-end collision	0.0015
H_{93}	Probability of leakage-fire caused by rear-end collision	0.0521
H_{94}	Probability of leakage-fire-explosion caused by rear-end collision	0.0193
H_{95}	Probability of fire caused by rear-end collision	0.0238
H_{96}	Probability of fire-explosion caused by rear-end collision	0.0104
H_{97}	Probability of fire-leakage caused by rear-end collision	0.0015
H_{98}	Probability of explosion caused by rear-end collision	0.0030
H_{99}	Probability of explosion-fire caused by rear-end collision	0.0045

Table S3. Different trigger factors in evacuation events lead to different event chain probabilities.

**Variable E_{ij}**	**Description**	**Probability value**
E_{11}	Evacuation probability of leakage caused by vehicle body fire	0.0000
E_{12}	Evacuation probability of leakage-explosion caused by vehicle body fire	0.0000
E_{13}	Evacuation probability of leakage-fire caused by vehicle body fire	0.0000

E_{14}	Evacuation probability of leakage-fire-explosion caused by vehicle body fire	0.0000
E_{15}	Evacuation probability of fire caused by vehicle body fire	0.0135
E_{16}	Evacuation probability of fire-explosion caused by vehicle body fire	0.0000
E_{17}	Evacuation probability of fire-leakage caused by vehicle body fire	0.0000
E_{18}	Evacuation probability of explosion caused by vehicle body fire	0.0068
E_{19}	Evacuation probability of explosion-fire caused by vehicle body fire	0.0000
E_{21}	Evacuation probability of leakage caused by valve failure	0.0338
E_{22}	Evacuation probability of leakage-explosion caused by valve failure	0.0000
E_{23}	Evacuation probability of leakage-fire caused by valve failure	0.0000
E_{24}	Evacuation probability of leakage-fire-explosion caused by valve failure	0.0000
E_{25}	Evacuation probability of fire caused by valve failure	0.0000
E_{26}	Evacuation probability of fire-explosion caused by valve failure	0.0000
E_{27}	Evacuation probability of fire-leakage caused by valve failure	0.0000
E_{28}	Evacuation probability of explosion caused by valve failure	0.0000
E_{29}	Evacuation probability of explosion-fire caused by valve failure	0.0000
E_{31}	Evacuation probability of leakage caused by tank rupture	0.0541
E_{32}	Evacuation probability of leakage-explosion caused by tank rupture	0.0000
E_{33}	Evacuation probability of leakage-fire caused by tank rupture	0.0000
E_{34}	Evacuation probability of leakage-fire-explosion caused by tank rupture	0.0000
E_{35}	Evacuation probability of fire caused by valve failure	0.0000
E_{36}	Evacuation probability of fire-explosion caused by tank rupture	0.0000
E_{37}	Evacuation probability of fire-leakage caused by tank rupture	0.0000
E_{38}	Evacuation probability of explosion caused by tank rupture	0.0000
E_{39}	Evacuation probability of explosion-fire caused by tank rupture	0.0000
E_{41}	Evacuation probability of leakage caused by brake failure	0.0203
E_{42}	Evacuation probability of leakage-explosion caused by brake failure	0.0000
E_{43}	Evacuation probability of leakage-fire caused by brake failure	0.0000
E_{44}	Evacuation probability of leakage-fire-explosion caused by brake failure	0.0000
E_{45}	Evacuation probability of fire caused by brake failure	0.0000
E_{46}	Evacuation probability of fire-explosion caused by brake failure	0.0000
E_{47}	Evacuation probability of fire-leakage caused by brake failure	0.0000
E_{48}	Evacuation probability of explosion caused by brake failure	0.0000
E_{49}	Evacuation probability of explosion-fire caused by brake failure	0.0000
E_{51}	Evacuation probability of leakage caused by tire burst	0.0203
E_{52}	Evacuation probability of leakage-explosion caused by tire burst	0.0000
E_{53}	Evacuation probability of leakage-fire caused by tire burst	0.0000
E_{54}	Evacuation probability of leakage-fire-explosion caused by tire burst	0.0000
E_{55}	Evacuation probability of fire caused by tire burst	0.0000
E_{56}	Evacuation probability of fire-explosion caused by tire burst	0.0000
E_{57}	Evacuation probability of fire-leakage caused by tire burst	0.0068
E_{58}	Evacuation probability of explosion caused by tire burst	0.0000
E_{59}	Evacuation probability of explosion-fire caused by tire burst	0.0000
E_{61}	Evacuation probability of leakage caused by rollover	0.3446
E_{62}	Evacuation probability of leakage-explosion caused by rollover	0.0000
E_{63}	Evacuation probability of leakage-fire caused by rollover	0.0068

E_{64}	Evacuation probability of leakage-fire-explosion caused by rollover	0.0068
E_{65}	Evacuation probability of fire caused by rollover	0.0000
E_{66}	Evacuation probability of fire-explosion caused by rollover	0.0000
E_{67}	Evacuation probability of fire-leakage caused by rollover	0.0000
E_{68}	Evacuation probability of explosion caused by rollover	0.0000
E_{69}	Evacuation probability of explosion-fire caused by rollover	0.0000
E_{71}	Evacuation probability of leakage caused by scraped	0.0338
E_{72}	Evacuation probability of leakage-explosion caused by scraped	0.0000
E_{73}	Evacuation probability of leakage-fire caused by scraped	0.0000
E_{74}	Evacuation probability of leakage-fire-explosion caused by scraped	0.0000
E_{75}	Evacuation probability of fire caused by scraped	0.0000
E_{76}	Evacuation probability of fire-explosion caused by scraped	0.0000
E_{77}	Evacuationprobability of fire-leakage caused by scraped	0.0000
E_{78}	Evacuation probability of explosion caused by scraped	0.0000
E_{79}	Evacuation probability of explosion-fire caused by scraped	0.0000
E_{81}	Evacuation probability of leakage caused by impact	0.1014
E_{82}	Evacuation probability of leakage-explosion caused by impact	0.0000
E_{83}	Evacuation probability of leakage-fire caused by impact	0.0135
E_{84}	Evacuation probability of leakage-fire-explosion caused by impact	0.0000
E_{85}	Evacuation probability of fire caused by impact	0.0000
E_{86}	Evacuation probability of fire-explosion caused by impact	0.0000
E_{87}	Evacuation probability of fire-leakage caused by impact	0.0000
E_{88}	Evacuation probability of explosion caused by impact	0.0000
E_{89}	Evacuation probability of explosion-fire caused by impact	0.0068
E_{91}	Evacuation probability of leakage caused by rear-end collision	0.1351
E_{92}	Evacuation probability of leakage-explosion caused by rear-end collision	0.0000
E_{93}	Evacuation probability of leakage-fire caused by rear-end collision	0.0068
E_{94}	Evacuation probability of leakage-fire-explosion caused by rear-end collision	0.0000
E_{95}	Evacuation probability of fire caused by rear-end collision	0.0068
E_{96}	Evacuation probability of fire-explosion caused by rear-end collision	0.0000
E_{97}	Evacuation probability of fire-leakage caused by rear-end collision	0.0000
E_{98}	Evacuation probability of explosion caused by rear-end collision	0.0000
E_{99}	Evacuation probability of explosion-fire caused by rear-end collision	0.0000
